# Urticating setae of tarantulas (Araneae: Theraphosidae): Morphology, revision of typology and terminology and implications for taxonomy

**DOI:** 10.1371/journal.pone.0224384

**Published:** 2019-11-11

**Authors:** Radan Kaderka, Jana Bulantová, Petr Heneberg, Milan Řezáč

**Affiliations:** 1 Faculty of Forestry and Wood Technology, Mendel University, Brno, Czechia; 2 Department of Parasitology, Faculty of Science, Charles University, Prague, Czechia; 3 Third Faculty of Medicine, Charles University, Prague, Czechia; 4 Biodiversity Lab, Crop Research Institute, Prague, Czechia; Nanjing Agricultural University, CHINA

## Abstract

Tarantula urticating setae are modified setae located on the abdomen or pedipalps, which represent an effective defensive mechanism against vertebrate or invertebrate predators and intruders. They are also useful taxonomic tools as morphological characters facilitating the classification of New World theraphosid spiders. In the present study, the morphology of urticating setae was studied on 144 taxa of New World theraphosids, including ontogenetic stages in chosen species, except for species with urticating setae of type VII. The typology of urticating setae was revised, and types I, III and IV were redescribed. The urticating setae in spiders with type I setae, which were originally among type III or were considered setae of intermediate morphology between types I and III, are newly considered to be ontogenetic derivatives of type I and are described as subtypes. Setae of intermediate morphology between that of body setae and type II urticating setae that were found in *Iridopelma hirsutum* and *Antillena rickwesti* may provide another evidence that type II urticating setae evolved from body setae. It is supposed that the fusion of barbs with the shaft may lead to the morphology of type II setae. As the type II setae of Aviculariinae evolved independently to the UrS of Theraphosinae and both subfamilies represent two non-sister groups, this should explain the differences in the morphology of body setae in Aviculariinae and Theraphosinae. The terminology of “barbs” and “reversed barbs” was revised and redefined, newly emphasizing the real direction of barbs.

## Introduction

The family Theraphosidae is the most species-rich and the most studied group of mygalomorph spiders [1]. An exclusive defensive mechanism using modified barbed setae called “urticating setae” (UrS) have been recorded in New World representatives belonging to subfamilies Theraphosinae Thorell, 1869 [2], Aviculariinae Simon, 1873 [3] and Psalmopoeinae Samm & Schmidt, 2010 [4] by many authors [5,6,7,8,9,10]. The UrS cover either the dorsal abdominal surface in Theraphosinae and Aviculariinae [6,7,8,10] or the prolateral face of the palpal femora in *Ephebopus* Simon, 1892 (Psalmopoeinae) [5]. When disturbed, spiders frequently disperse their abdominal UrS by moving the posterior legs, or, in the case of palpal setae, by moving the palps along the basal segments of chelicerae and directing these airborne setae towards the intruder [5,6]. Some representatives of Aviculariinae (*Avicularia* Lamarc, 1818, *Iridopelma* Pocock, 1901 and *Pachistopelma* Pocock, 1901) and probably also *Kankuamo* Perafán et al., 2016 (Theraphosinae) require direct contact with the intruder to release the UrS [7,8,11]. The UrS penetrate the attacker’s skin or mucous membranes, whereby inducing a physical irritation. Some Theraphosinae also use the UrS as an additional component for making egg sacs and the silk mat for moulting as a passive defensive strategy against ants or the larvae of phorid flies [8,9,12].

**Fig 1:**
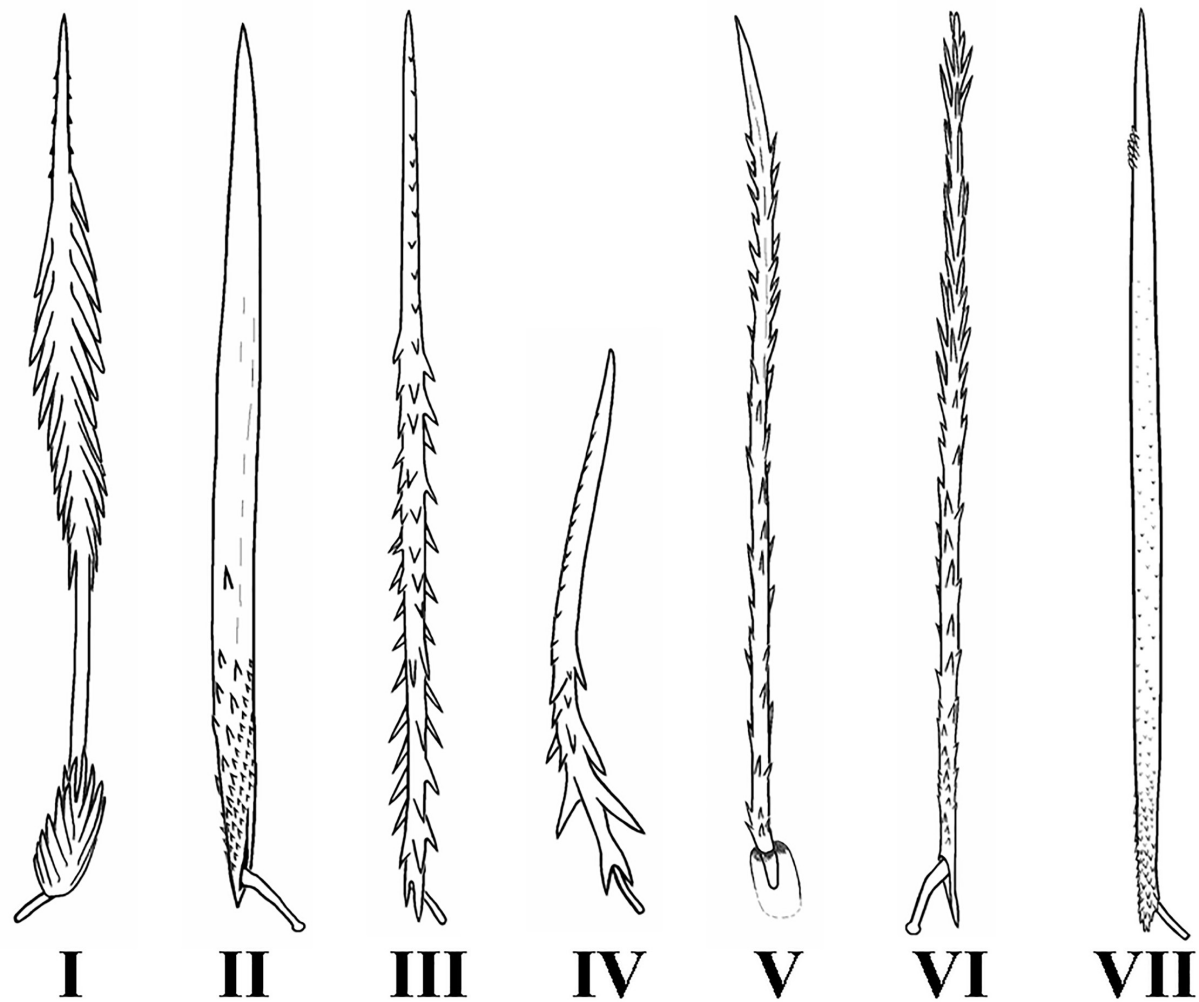
Abdominal urticating setae of types I, II, III, IV, VI and VII with their supporting stalks, and palpal urticating setae of the type V with the insertion socket.

To date, seven morphological types of UrS have been described (Fig 1). They differ in location, shape, size and orientation of barbs along the shaft, and the length/width ratio. Types I–IV were described and illustrated for the first time by Cooke et al. [6], who noted their value in the systematics of Theraphosidae. Later, Marshall & Uetz [5] described palpal type V in *Ephebopus*, and Pérez-Miles [13] proposed type VI in the Mexican genus *Hemirrhagus* Simon, 1903. Most recently, Perafán et al. [11] described the UrS of type VII in the Colombian genus *Kankuamo*. The occurrence of UrS types in South American genera of Aviculariinae, Psalmopoeinae and Theraphosinae is presented in Table 1.

**Table 1:** Distribution of urticating setae types according to the previously published papers or unpublished observations.

	URTICATING SETAE TYPES							
Genus	I	II	III	IV	V	VI	VII	References
AVICULARIINAE								
*Antillena* Fukushima & Bertani, 2017	–	+	–	–	–	–	–	[14]
*Avicularia* Lamarck, 1818	–	+	–	–	–	–	–	[15]
*Caribena* Fukushima & Bertani, 2017	–	+	–	–	–	–	–	[14]
*Iridopelma* Pocock, 1901	–	+	–	–	–	–	–	[15]
*Pachistopelma* Pocock, 1901	–	+	–	–	–	–	–	[15]
*Typhochlaena* C. L. Koch, 1850	–	+	–	–	–	–	–	[15]
*Ybyrapora* Fukushima & Bertani, 2017	–	+	–	–	–	–	–	[14]
PSALMOPOEINAE								
*Ephebopus* Simon, 1892	–	–	–	–	+	–	–	[5]
*Psalmopoeus* Pocock, 1895	–	–	–	–	–	–	–	[16]
*Pseudoclamoris* Hüsser, 2018	–	–	–	–	–	–	–	[17]
*Tapinauchenius* Ausserer, 1871	–	–	–	–	–	–	–	[6,16]
THERAPHOSINAE								
*Acanthoscurria* Ausserer, 1871	+	–	+	–	–	–	–	[18]
*Acentropelma* Pocock, 1901 ^a^	+	–	?	–	–	–	–	[19,20]
*Aenigmarachne* Schmidt, 2005	–	–	–	–	–	+	–	[21]
*Agnostopelma* Pérez-Miles & Weinmann, 2010	–	–	+	+	–	–	–	[22]
*Aguapanela* Perafán & Cifuentes, 2015	–	–	+	+	–	–	–	[23]
*Aphonopelma* Pocock, 1901	+	–	–	–	–	–	–	[6,18,24]
*Bistriopelma* Kaderka, 2015	–	–	+	–	–	–	–	[25]
*Bonnetina* Vol, 2000	–	–	+	–	–	–	–	[26]
*Brachypelma* Simon, 1891	+	–	+	–	–	–	–	[18]
*Bumba* Pérez-Miles, Bonaldo & Miglio, 2014	–	–	+	+	–	–	–	[27]
*Cardiopelma* Vol, 1999	?	?	+	?	?	?	?	J. I. Mendoza Marroquín, pers commun.
*Catanduba* Yamamoto et al., 2012	–	–	+	–	–	–	–	[28]
*Chromatopelma* Schmidt, 1995	–	–	+	–	–	–	–	[16]
*Citharacanthus* Pocock, 1901	+	–	–	–	–	–	–	[18]
*Clavopelma* Chamberlin, 1940	+	–	–	–	–	–	–	[16]
*Cotztetlana* Mendoza Marroquín, 2012	+	–	–	–	–	–	–	[29]
*Crassicrus* Reichling & West, 1996	+	–	–	–	–	–	–	[16]
*Cubanana* Ortiz, 2008	+	–	+	–	–	–	–	[30]
*Cyclosternum* Ausserer, 1871	–	–	+	–	–	–	–	[18]
*Cyriocosmus* Simon, 1903	–	–	+	–	–	–	–	[31,32]
*Cyrtopholis* Simon, 1892	+	–	+	–	–	–	–	types I, III [6]; type III [16]; type I [18]
*Davus* Cambridge, 1892	–	–	+	–	–	–	–	[16,19]
*Euathlus* Ausserer, 1875	–	–	+	+	–	–	–	[33]
*Eupalaestrus* Pocock, 1901	+	–	+	–	–	–	–	[18]
*Eurypelmella* Strand, 1907 ^a^	+	–	?	–	–	–	–	[19,20]
*Grammostola* Simon, 1892	–	–	+	+	–	–	–	[6]; as *Phrixotrichus* in [18]
*Hapalopus* Ausserer, 1875 ^b^	–	–	+	+	–	–	–	[18], [31]
*Hapalotremus* Simon, 1903	–	–	+	–	–	–	–	[18]
*Hemirrhagus* Simon, 1903	–	–	–	–	–	+	–	[13,34,35]
*Homoeomma* Ausserer, 1871	–	–	+	+	–	–	–	[18]
*Kankuamo* Perafán et. al., 2016	–	–	–	–	–	–	+	[11]
*Kochiana* Fukushima et al., 2008	–	–	+	–	–	–	–	[36]
*Lasiodora* C. L. Koch, 1850	+	–	+	–	–	–	–	[18]
*Lasiodorides* Schmidt & Bischoff, 1997	+	–	+	–	–	–	–	[16]
*Longilyra* Gabriel, 2014	+	–	+	–	–	–	–	[37]
*Magnacarina* Mendoza et al., 2016	–	–	+	–	–	–	–	[38]
*Magulla* Indicatti et al., 2008	–	–	+	+	–	–	–	[39]
*Megaphobema* Pocock, 1901	+	–	+	–	–	–	–	[18]
*Melloleitaoina* Gerschman & Schiapelli, 1960	–	–	+	+	–	–	–	[18,40]
*Metriopelma* Becker, 1878 ^c^	+	–	–	–	–	–	–	A. Locht, pers. commun.; R. Gabriel, pers. commun.
*Miaschistopus* Pocock, 1897 ^a^	+	–	?	–	–	–	–	[19,20]
*Munduruku* Miglio et al., 2013	–	–	+	+	–	–	–	[41]
*Mygalarachne* Ausserer, 1871	+	–	+	–	–	–	–	[42]
*Neischnocolus* Petrunkevitch, 1925	+	–	–	–	–	–	–	[43,44]
*Neostenotarsus* Tesmoingt & Schmidt, 2002	+	–	+	–	–	–	–	[45]
*Nesipelma* Schmidt & Kovařík, 1996	+	–	–	–	–	–	–	[16,46]
*Nhandu* Lucas, 1981	+	–	+	–	–	–	–	[18]
*Pamphobeteus* Pocock, 1901	+	–	+	–	–	–	–	[18]
*Phormictopus* Pocock, 1901	+	–	+	–	–	–	–	[18]
*Phrixotrichus* Simon, 1889	–	–	+	+	–	–	–	[33]
*Plesiopelma* Pocock, 1901	–	–	+	+	–	–	–	[18]
*Proshapalopus* Mello-Leitão, 1923	+	–	+	–	–	–	–	[47]
*Pseudhapalopus* Strand, 1907	+	–	+	–	–	–	–	[16]
*Pterinopelma* Pocock, 1901	+	–	+	–	–	–	–	[48]
*Reversopelma* Schmidt, 2001	+	–	+	–	–	–	–	[49]
*Schizopelma* Cambridge, 1897	–	–	+	–	–	–	–	[18]
*Scopelobates* Simon, 1903	+	–	–	–	–	–	–	[50]
*Sericopelma* Ausserer, 1875	+	–	+	–	–	–	–	[18]
*Sphaerobothria* Karsch, 1879	+	–	–	–	–	–	–	[18]
*Stichoplastoris* Rudloff, 1997	+	–	–	–	–	–	–	[16]
*Theraphosa* Thorell, 1870	–	–	+	–	–	–	–	[18]
*Thrixopelma* Schmidt, 1994	–	–	+	+	–	–	–	[16]
*Tmesiphantes* Simon, 1892	–	–	+	+	–	–	–	type III [18]; types III, IV [51]
*Umbyquyra* Gargiulo, Brecovit, Lucas, 2018	+	–	+	–	–	–	–	[52]
*Vitalius* Lucas et al., 1993	+	–	+	–	–	–	–	types I, III [6]; type I [16,18]
*Xenesthis* Simon, 1891	+	–	–	–	–	–	–	[18]

Legend: “+” = present; “–” = absent; “?” = unknown. Classification of urticating setae types follows Cooke et al. [6], Marshall & Uetz [5], Pérez-Miles [13] and Perafán et al. [11].

^a^ The genera *Miaschistopus* Pocock, 1897, *Acentropelma* Pocock, 1901 and *Eurypelmella* Strand, 1907 were restored by Gabriel [19], but these nomenclatural acts were not accompanied by the generic diagnoses and the redescriptions of type species. The cited paper only referred to any prepared article focused on these genera. As the presence / absence of urticating setae was unknown until now, the cited genera were provisionally listed in Theraphosinae according to Turner et al. [20].

^b^ In *Hapalopus* Ausserer, 1875, type IV setae are present only in *Hapalopus butantan* (Pérez-Miles, 1998) [31].

^c^ Based on the topotype of *Metriopelma breyeri* Becker, 1878 from Guanajuato, Mexico (in BMNH). The holotype is lost.

Some species from the subfamily Theraphosinae have more than one type of UrS and setae of intermediate morphology [10,53]. Types I or IV usually occur in combination with type III [10]. The co-occurrence of types I and IV has not been recorded, nor have any intermediates between type II and types I, III or IV ([16]; personal observation). Studying the ontogeny of five Uruguayan species, Pérez-Miles [10] found that type III occurred later than the other types present (I or IV). He hypothesised that type III setae represent two different types of setae masked by morphological similarity and derived from type I and IV, respectively. Pérez-Miles [10] also found some differences in the morphology of type III setae in species with the co-occurrence of types I+III and III+IV: the basal end of the type III seta in specimens having types I+III has a broad shaft, and at high magnification, this region shows flattened barbs. In specimens with types III+IV, the basal end of the type III seta has no flattened barbs and the shaft is not extended to the tip.

Bertani & Marques [7] and Bertani & Guadanucci [9,53] suggested that the UrS of types II, V and III had evolved independently and that the types I and IV had evolved later from an ancestor type III setae. This hypothesis was supported by the differences in position, structure and the release mechanism of UrS and by the existence of intermediates between types I and III, and III and IV. However, no intermediates between types I and IV were found. Bertani & Guadanucci [9] hypothesised that urticating setae of type II and type III had independently evolved from the relevant body setae and noted 1) the identical manner in which they are inserted into the spider tegument, 2) the resemblance between the truncated basal part of body setae and stalks in UrS, and 3) the morphological similarity of basal barbs in some variants of body setae and UrS. They found body setae setae variants with either type II or III urticating setae, but not with types I and IV [9]. A reconstruction of phylogenetic relationships based on analysis of set of genes [54] confirmed that urticating setae of type II and type III had independently evolved.

Since Pérez-Miles et al. [18] used UrS types in the phylogenetic analysis of Theraphosinae, the typology of the UrS has become an important taxonomical tool. The monophyly of Theraphosinae was reinforced by the presence of the abdominal UrS of types I, III and/or IV [18] and type VI and VII [11]. Bertani & Marques [7] considered the absence of UrS in the majority of Theraphosidae as a plesiomorphic state.

The aim of this study is 1) to revise the morphological types or subtypes of UrS based on the examination of 144 species of Theraphosidae representing all known South American subfamilies, including their development during ontogeny in chosen species, and also 2) to implicate new findings for taxonomy of Theraphosidae with a support of previously published phylogenetic analyses based on both morphological and molecular data. The aim of the revision is 3) also to critically evaluate what is known in this field of research and 4) to compare the evolutionary hypotheses of UrS development derived from the mentioned phylogenetic analyses.

## Materials and methods

**Fig 2:**
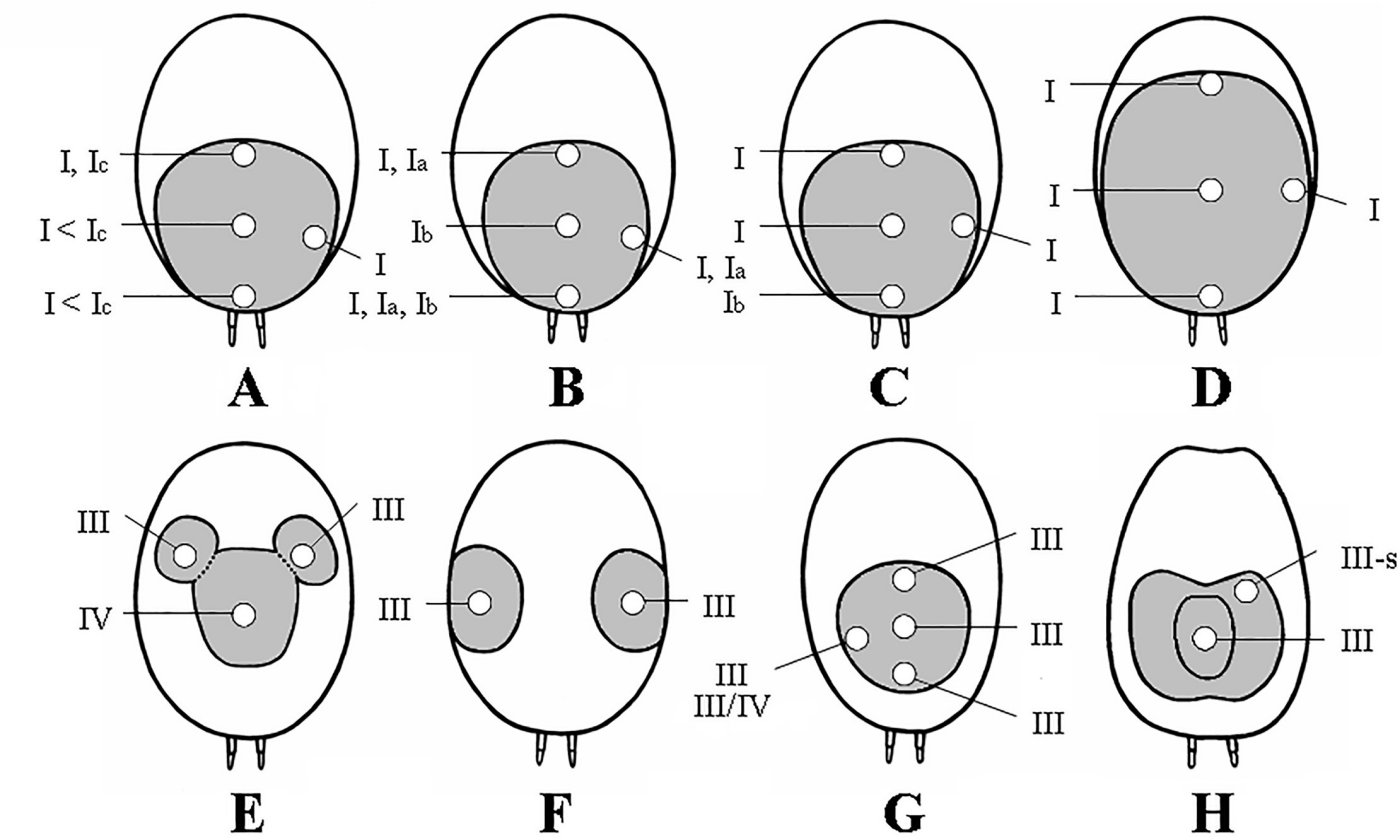
Distribution of urticating setae types (grey zones) on the dorsal abdomen of particular Theraphosinae. (A) *Eupalaestrus larae*, female from Argentina. (B) *Cyrtopholis* sp., male from Cuba, Guantánamo. (C) *Phormictopus auratus*, female from Cuba, Holguín province. (D) *Aphonopelma crinirufum*, female from Costa Rica, Puntarenas. (E) *Magulla obesa*, male (according to [39]). (F) *Phrixotrichus vulpinus*, male from Chile. (G) *Chromatopelma cyanopubescens*, male from Venezuela. (H) *Kochiana brunnipes*, female. Abbreviations: III/IV = urticating setae of intermediate morphology between types III and IV; III-s = short type III setae, length 0.07–0.08. White circles represent the spots of sampling.

For the morphological analyses, the UrS were removed from four different areas (anterior, central, posterior and lateral, see Fig 2) using forceps, placed in alcohol, and subsequently examined and measured using an Olympus BH2-RFCA binocular microscope. The samples for measurement were taken from the central area of the urticating setae patch only. The setae without supporting stalks were measured along the shaft and the seta curvature was interpolated. After the collection of each sample, the forceps were cleaned to avoid contamination by the setae from the previous sample. After dehydration in a CPD 030 critical point dryer (BAL-TEC GmbH, Schalkmühle, Germany) and gold coating in a SCD 030 ion sputtering device (BAL-TEC GmbH, Schalkmühle, Germany), the selected samples were examined using a JSM-6380LV scanning electron microscope (JEOL Ltd., Akishima-shi, Japan). The UrS barbs were measured in the basal third of the seta. The ontogenetic stage was characterised via the carapace length, which was measured using a calliper. All of the measurements were provided in millimetres. For the material used in morphological studies, see S1 File. The typology of the UrS follows Cooke et al. [6] (in part), Marshall & Uetz [5], Pérez-Miles [13] and Perafán et al. [11]. For morphological repetitions of the basic types of urticating setae we used the term “subtype”. The concept of “barbs” and “reversed barbs” proposed by Cooke et al. [6] was revised and unified, newly emphasizing a real direction of barbs. Generally, setal barbs, which point upwards to the apical tip and which are present in the majority of covering setae of spiders, are called “barbs”. The setal barbs pointing upwards are present, for example, in plumose setae or stout plumose stridulatory setae or bristles, tarsal scopula setae, long tactile setae, any types of urticating setae or in body setae from which urticating setae probably evolved in Aviculariinae and Theraphosinae (Mygalomorphae: Theraphosidae). Barbed setae (sicate, pinnate or arborate setae) is also known, for example, in Gnaphosidae (Araneomorphae) [55]. The barbs of the opposite direction pointing downward to the setal base, are called “reversed barbs” in this study. As they are present only in a few types of UrS, they are considered a derived character.

The terminology of barbs and reversed barbs was herein unified for all setal structures and in the case of type I setae changed for the reasons mentioned below. Cooke at al. [6] described the morphology of type I setae for the first time, including the terms “barbs” and “reversed barbs”. The most extended barbs of the central area (midsection) were called “main barbs” and the basal barbs of the opposite direction were called “reversed barbs”. To put simply, whatever barbs of the opposite direction to the main barbs might be called “reversed barbs”. The term “main barbs” was used by Cooke et al. [6] only for type I setae and this concept of “main barbs” and “reversed barbs” was followed by Perafán et al. [11] in the description of type VII setae in *Kankuamo*. In types III, IV, and VII, the main barbs are of the same direction to the main barbs in type I, pointing downwards to the basal end of the seta, but in opposite direction to the main barbs of type II, V, and VI, whose barbs are pointing upwards to the apical end. To unify the terminology in all setal structures, we suggest the above mentioned definitions taking into account especially the real direction of barbs. This new concept allows descriptions of all setal types to be unified and mutually comparable in comparison with Cooke et al. [6], whose concept of “barbs” and “reversed barbs” does not allow this.

Consequently, we renamed section C (= basal barbs) and section B (central section without barbs) (according to Cooke et al. [6]) to section B (= basal barbs) and central section C2 (central section without barbs). The section C2 of main barbs corresponds with main barbs according to Cooke et al. [6]. According to Cooke et al. [6], sections “A”, “B” and “C” refer to the measurements in Tables 2–4 (to single columns), and they were not a part of the description of type I setae. We are persuaded that these minor changes in the amended terminology and abbreviations of type I UrS sections do not cause any confusion among other researchers. In this case we recommend to use the non-abbreviated versions referring to single setal sections.

**Table 2:** Length ranges of basic type I setae.

Species	Sex	Length of setae
*Acanthoscurria* sp. from Paraguay	♀	0.40–0.52
*Aphonopelma seemanni*	♀	0.39–0.41
*Brachypelma klaasi*	♀	0.37–0.43
*Eupalaestrus weijenberghi*	♀	0.27–0.37
*Cyclosternum schmardae*	♀	0.52–0.60
*Crassicrus lamanai*	♀	0.35–0.37
*Phormictopus auratus*	♀	0.21–0.28
*Reversopelma petersi*	♀	0.40–0.53

**Table 3:** Length ranges of subtype I_a_ setae.

Species	Sex	Length of setae
*Aphonopelma bicoloratum*	♂	0.37–0.42
*Aphonopelma seemanni*	♂	0.40
*Brachypelma klaasi*	♀	0.46–0.57
*Brachypelma verdezi*	♀	0.61–0.67
*Cyrtopholis* sp. from Cuba, Santiago de Cuba province	♂	0.43
*Megaphobema mesomelas*	♂	0.55–0.62
*Sericopelma melanotarsum*	♀	0.57–0.65
*Sphaerobothria hoffmanni*	♂	0.55–0.57

**Table 4:** Length ranges of subtype I_b_ setae.

Species	Sex	Length of setae
*Aenigmarachne sinapophysis*	♂	0.27–0.31
*Aphonopelma seemanni*	♂	0.58
*Brachypelma verdezi*	♂	0.58–0.60
*Brachypelma verdezi*	♀	0.45–0.61
*Cyrtopholis* sp. from Cuba, Santiago de Cuba province	♂	0.42–0.46
*Metriopelma* sp. from Venezuela, Isla Margarita	♂	0.35–0.41
*Neischnocolus* sp. from Ecuador	♀	0.30–0.37
*Phormictopus auratus*	♀	0.53–0.63

Juveniles were identified according to the morphological characters of their parental pair or the characters after maturation. The designation of postembryonic stages followed Foelix [56]. The phylogenetic concept of Theraphosinae, Aviculariinae, Psalmopoeinae and Stromatopelminae follows Turner et al. [20], Lüddecke et al. [54] and Hüsser [17]. The nomenclature followed the World Spider Catalog [1], with the following exceptions: the genus *Metriopelma* (see remark 2 at the end of the chapter Descriptions of basic urticating setae types and their subtypes) is thus far insufficiently diagnosed, and its revision is needed, together with the species provisionally presented here in that genus.

## Results

### Distribution of UrS on the abdominal surface in Theraphosinae

The dorsal abdominal cuticle of Theraphosinae is covered with UrS varying in the shape, size and even in the area number as some taxa may have two separate lateral areas of UrS, e.g., *Bistriopelma* Kaderka, 2015 and *Phrixotrichus* Simon, 1889, instead of one dorsal area. Generally, the taxa with UrS of types III, IV and III+IV show higher variability in the shape, size and number of urticating setae patches, in comparison to the taxa with type I setae arranged in one dorsal patch (Fig 2; Bertani & Guadanucci [9]: Figs 5–12). Theraphosinae with type I setae may have only one to two patterns and differ from the taxa with types III, IV or III+IV, in which another seven patterns were recognised [9]. The theraphosine genus *Magulla* Simon 1892 displays another pattern, which consists of type IV setae placed in the central dorsal patch and type III setae present in two separate anterior patches connected by the central patch [39].

### Descriptions of basic urticating setae types and their subtypes

Cooke et al. [6] defined type III setae as 0.3–1.2 mm long setae with thin straight shaft, fine point, barbs along at least one-half the length and considerable variation not only in length but also in the size and density of barbs among setae of this type. As the arrangement of barbs in two opposite rows in *Acanthoscurria rhodothele* Mello-Leitão, 1923 and in the silhoutte of type III setae presented by Cooke et al. [6] is typical for type I setae and because Pérez-Miles [10] found some differences in the morphology of type III setae in species with the co-occurrence of types I+III and III+IV, we decided to revise and redefine type III setae.

We suggest a new terminology for type I setae with the phylogenetic support of the molecular analyses carried out by Turner et al. [20] and Lüddecke et al. [54], showing taxa with type I setae as monophyletic group, and supported by the findings that were demonstrated in the present study in UrS development during ontogeny in 14 species of Theraphosinae. We consider the UrS that were formerly classified as type III or were considered setae of intermediate morphology between those of types I and III in species possessing both of these types to be ontogenetic derivatives of type I setae as they develop later in the ontogenetic development and they are derived from type I seta morphology. They are further described as subtypes of type I. Other than that, the typology of UrS follows Cooke et al. [6], Marshall & Uetz [5], Pérez-Miles [13] and Perafán et al. [11].

**Fig 3:**
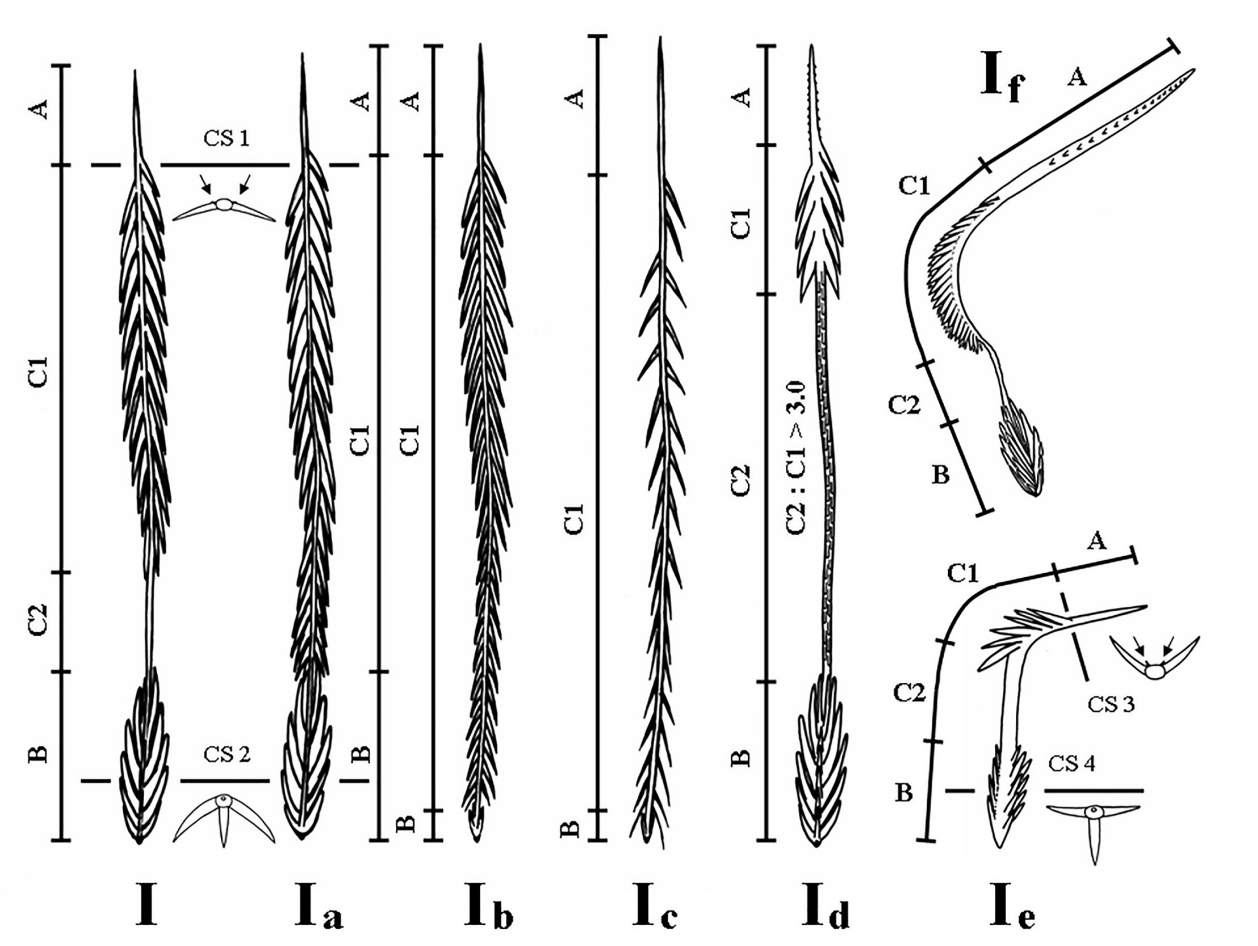
Urticating setae of type I and its subtypes I_a_, I_b_, I_c_, I_d_, I_e_ and I_f_. Cross sections (CS) 1–4: arrangement of barbs in cross-sections. The arrows show two rows of opposite reversed denticles on the apical end of section C1. Abbreviations: B = basal section; C1 = central section with well-developed reversed barbs (corresponds with “main barbs” according to Cooke et al. [6]); C2 = central section, which is bare or with two parallel longitudinal rows of short confluent reversed barbs or denticles; A = apical section.

**Fig 4:**
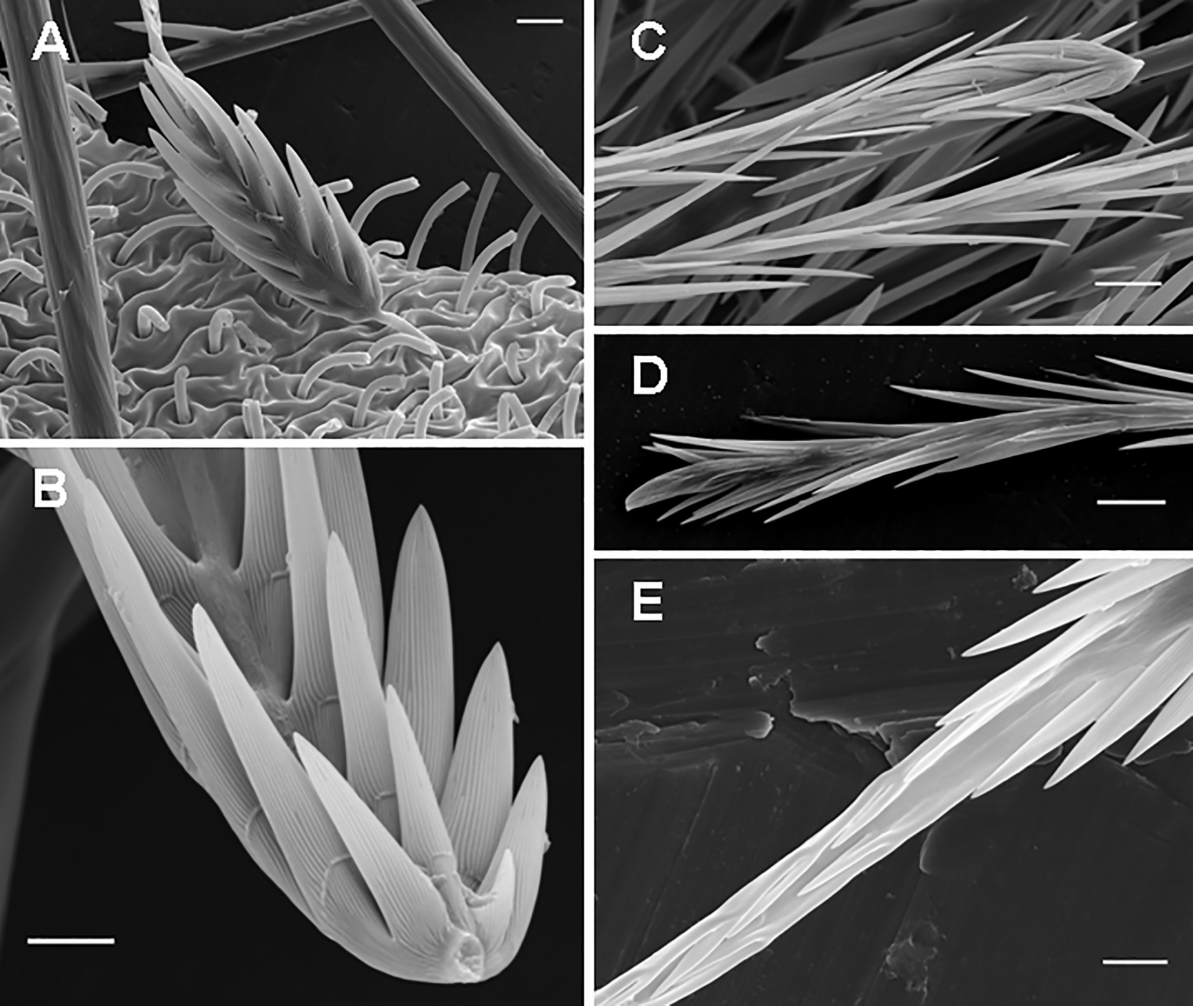
Urticating setae of *Brachypelma smithi*, immature male. (A) Basic type I, basal section, connected to the dorsal abdominal surface by a supporting stalk and surrounded by other supporting stalks without urticating setae. Scale bar = 10 μm. (B) Basic type I, basal section with three rows of barbs. Scale bar = 5 μm. (C) Type I, subtypes I_c_, midsection (below) and basal section (above) with basal barbs reduced in both number and size. Scale bar = 10 μm. (D) Type I, subtype I_c_, basal section with strongly reduced basal barbs. Scale bar = 10 μm. (E) Basic type I, detail of the midsection with two parallel longitudinal rows of short, confluent reversed barbs in the left lower part of the figure. Scale bar = 5 μm.

**Type I UrS**, including their subtypes as morphological repetitions (Fig 3), are generally characterised by the presence of reversed barbs in the midsection and a broad basal end. The basal barbs are present and may be developed (Fig 4A and 4B), reduced (Fig 4C) or strongly reduced (Fig 4D). The connection of the UrS with a supporting stalk, which is a so-called break-off zone, is beneath the basal section of non-reversed barbs; the one exception is in subtype I_f_ with an additional break-off zone between sections B and C1.

**Basic type I** (Figs 1, 3, 4A, 4B and 4E; Table 2): it is characterised by a shaft with two axial flections and four axial sections (from the basal to the apical end): the basal barbs (section B) are arranged in two opposite, concavely arranged rows; in addition, there is a central third row of longitudinally arranged barbs. The following section (section C2, see Fig 3), which was formerly described as a bare shaft [6], has two parallel rows of short confluent reversed barbs or denticles in adults (Fig 4E). This section may be bare in the first nymphal stages. The following section (section C1, see Fig 3) carries two opposite rows of reversed barbs, which can be arranged helically, in *Reversopelma petersi* Schmidt, 2001, with 1–4 distal pairs of non-reversed barbs. The tapering apical section (section A, see Fig 3) has only two rows of small reversed denticles. Length of setae: 0.21–0.60.

**Fig 5:**
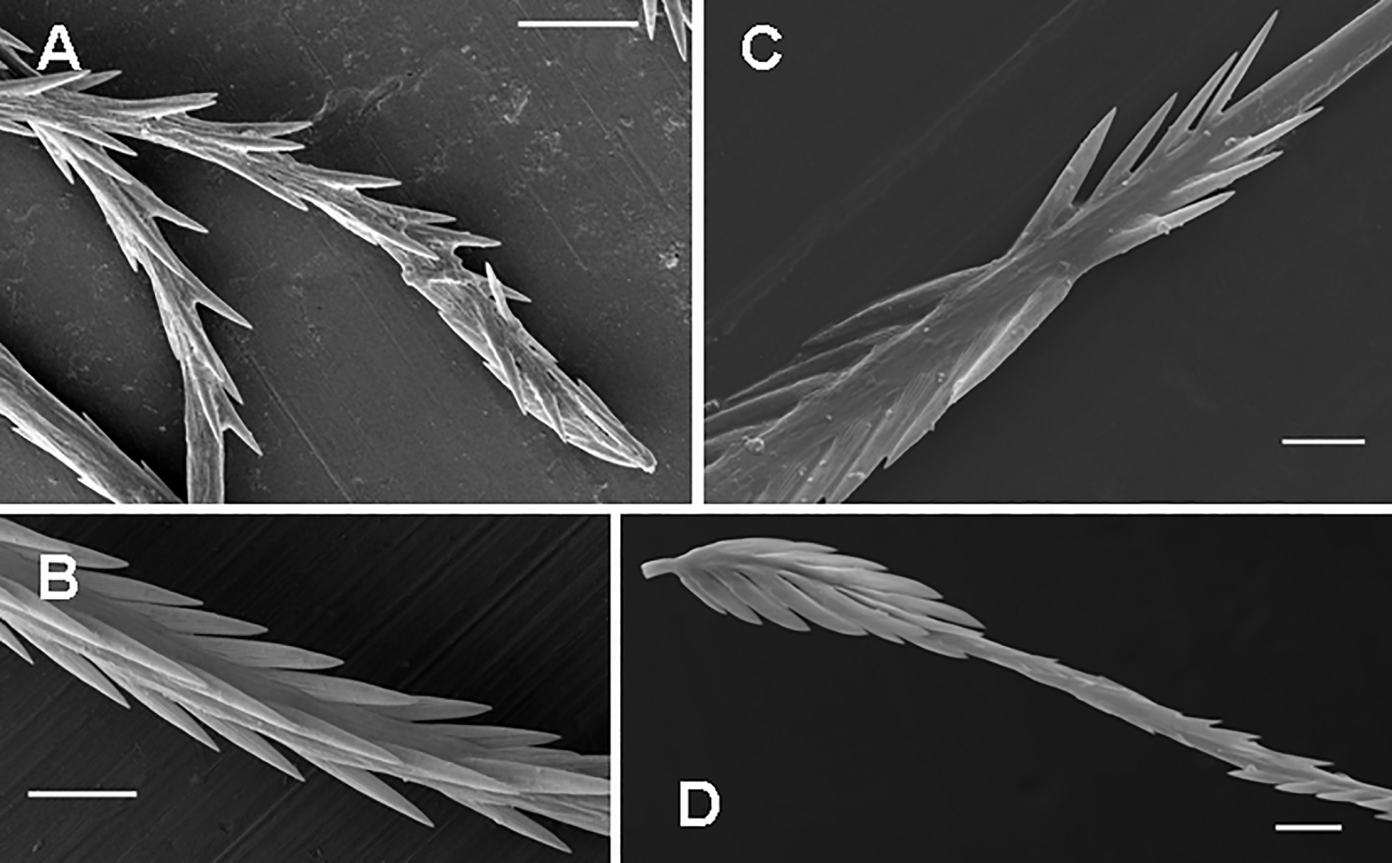
Urticating setae of type I and its subtypes I_b_ and I_d_. (A) *Aenigmarachne sinapophysis*, male holotype. Type I, subtype I_b_, basal section with barbs reduced both in number and size. (B) *Aphonopelma seemanni*, male. Type I, subtype I_b_, midsection with three rows of reversed barbs. (C) *Reversopelma petersi*, male. Type I, distal end of the midsection (section C1) with a few pairs of non-reversed barbs. (D) *Citharacanthus longipes*, female. Type I, subtype I_d_, detail of the basal section and section C2. Scale bar = 10 μm.

**Subtype I**_**a**_ (Fig 3; Bertani [47]: Fig 5; Table 3): it differs from the basic type I in the absence of the section C2; a third central row of basal barbs or denticles is present. Length of setae: 0.37–0.67. Length of reversed barbs: 0.005–0.030.

**Subtype I**_**b**_ (Figs 3, 5A and 5B; Table 4): it is characterised by a nearly straight shaft or a shaft with one axial flection and by three axial sections (from the basal to the apical end): the reduced basal section (section B) carries two opposite rows of barbs that are reduced in length and confluent with the shaft, or the barbs are absent. Section C2 is absent. Two rows of opposite reversed barbs on section C1 can be complemented by a third row in the proximal part. The tapering apical section (section A) carries denticles arranged in two opposite rows. Length of setae: 0.27–0.63. Length of reversed barbs: 0.007–0.011.

**Fig 6:**
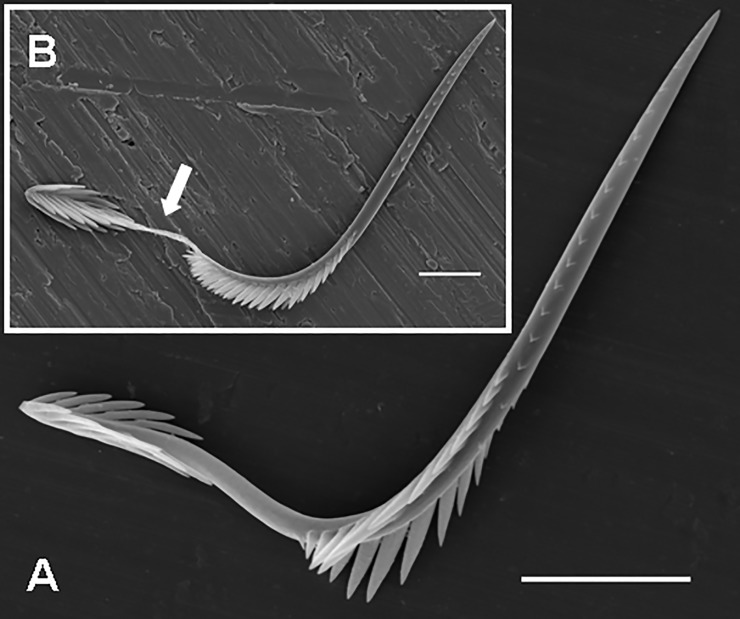
Urticating setae of type I and its subtypes I_e_ and I_f_. (A) *Metriopelma* sp., female from Margarita Island, Venezuela. Type I, subtype I_e_, midsection C1 with barbs reduced both in length and number, midsection C2 completely bare, smooth and thicker than in the subtype I_f_. (B) *Pseudhapalopus* sp., male from Colombia. Type I, subtype I_f_, with the midsection C2 narrow and flattened, with a rough surface. Section C2 represents a zone in which the seta breaks off. Scale bar = 20 μm.

**Subtype I**_**c**_ (Figs 3, 4C and 4D; Pérez-Miles [10]: Fig 6, basal section; Table 5): it is characterised by a long, almost straight shaft or a shaft with one axial flection and three axial sections (from the basal to the apical end): the reduced basal section B carries short barbs, which strongly adhere to the shaft, or the barbs are absent. Section C2 is absent. The following central section, C1, carries thin and long reversed barbs, which are less dense than in the other subtypes, arranged in two longitudinal rows, and complemented by additional one or two rows in the proximal part only. The tapering apical section (section A) carries reversed denticles arranged in two opposite rows. This subtype is clearly longer than the other type I subtypes. Subtype I_c_ differs from I_b_ in total length (I_c_ > I_b_), lower density of barbs and larger size (in I_c_ 2–6 times as long as in I_b_). It was formerly misinterpreted as a type III seta due to the morphological resemblance, but it differs in the incrassate shaft at the basal end, which carries flattened barbs visible at high magnification (approximately 2000×) [10]. This subtype always occurs with basic type I or another subtype of the type I seta. Length of setae: 0.52–1.64. Length of reversed barbs: 0.022–0.050.

**Table 5:** Length ranges of subtype I_c_ setae.

Species	Sex	Length of setae
*Acanthoscurria geniculata*	♀	0.67–0.87
*Acanthoscurria suina*	♂	0.58–0.67
*Brachypelma auratum*	♀	1.10–1.27
*Brachypelma baumgarteni*	♀	1.56–1.64
*Brachypelma klaasi*	♀	0.81–0.98
*Brachypelma albiceps*	♀	0.73–0.81
*Eupalaestrus larae*	♀	0.66–0.74
*Nhandu tripepii*	♀	0.71–0.75
*Phormictopus auratus*	♀	0.52–0.58
*Phormictopus cubensis*	♀	0.61–0.87
*Phormictopus* sp. from Cuba, Guanabo	♀	0.73–0.84
*Sericopelma melanotarsum*	♀	0.73–0.75

**Subtype I**_**d**_ (Figs 3 and 5D; Table 6): it is characterised by a shaft with one axial flection and four axial sections (from the basal to the apical end): the basal barbs (section B) are arranged in two opposite, concavely arranged rows, with a central third row of longitudinal barbs. The following section (section C2), has two parallel rows of short confluent reversed barbs or denticles in adults. It is unusually long, with the C2/C1 ratio higher than 3.0 (up to 9.0). Section C1 is basally flexed, with two opposite rows of reversed barbs. Length of setae: 0.31–0.77. This subtype was found in the genera *Citharacanthus* Pocock, 1901 and *Neischnocolus* Petrunkevitch, 1925.

**Table 6:** Length ranges of subtype I_d_ setae.

Species	Sex	Length of setae
*Citharacanthus longipes*	♀	0.51–0.56
*Neischnocolus* sp. from Costa Rica	♂	0.64–0.77
*Neischnocolus* sp. from Venezuela	♀	0.31–0.68
*Neischnocolus weinmanni*	♂	0.59

**Subtype I**_**e**_ (Figs 3 and 6A; Table 7): it is characterised by its very small size. The setae have one axial flection and four axial sections (from the basal to the apical end): the section of basal barbs (section B) carries three rows of barbs that have a T-shape in cross-section. The following cylindrical C2 section is smooth and stout in comparison to the I_f_ subtype. The following short C1 section has two opposite rows of reversed barbs and the tapering apical section (section A) carries two opposite rows of small reversed denticles. Length of setae: 0.11–0.15. Length of reversed barbs: up to 0.011. This subtype was found only in the females of *Metriopelma* sp. from Margarita Island, Venezuela.

**Table 7:** Length ranges of subtype I_e_ setae.

Species	Sex	Length of setae
*Metriopelma* sp. from Venezuela, Isla Margarita	♀	0.11–0.15

**Subtype I**_**f**_ (Figs 3 and 6B; Table 8): it is characterised by a smaller size and an extremely narrow and flattened shaft in section C2, which has a rough surface. The setae have two axial flections and four axial sections (from the basal to the apical end): section B is arranged in two opposite rows, and the third central row of longitudinal basal barbs is present. Section C2 represents a zone in which the distal part of the seta breaks off even more easily than the seta breaks off from a supporting stalk. The following section, C1, is curved and has two opposite rows of reversed barbs; the tapering apical section (section A) has two opposite rows of reversed denticles. Length of setae: 0.18–0.21. Length of reversed barbs: up to 0.011. This subtype was found only in a juvenile male of *Pseudhapalopus* sp. from Colombia.

**Table 8:** Length ranges of subtype I_f_ setae. juv. = juvenile specimen.

Species	Sex	Length of setae
*Pseudhapalopus* sp. from Colombia	juv. ♂	0.18–0.21

**Fig 7:**
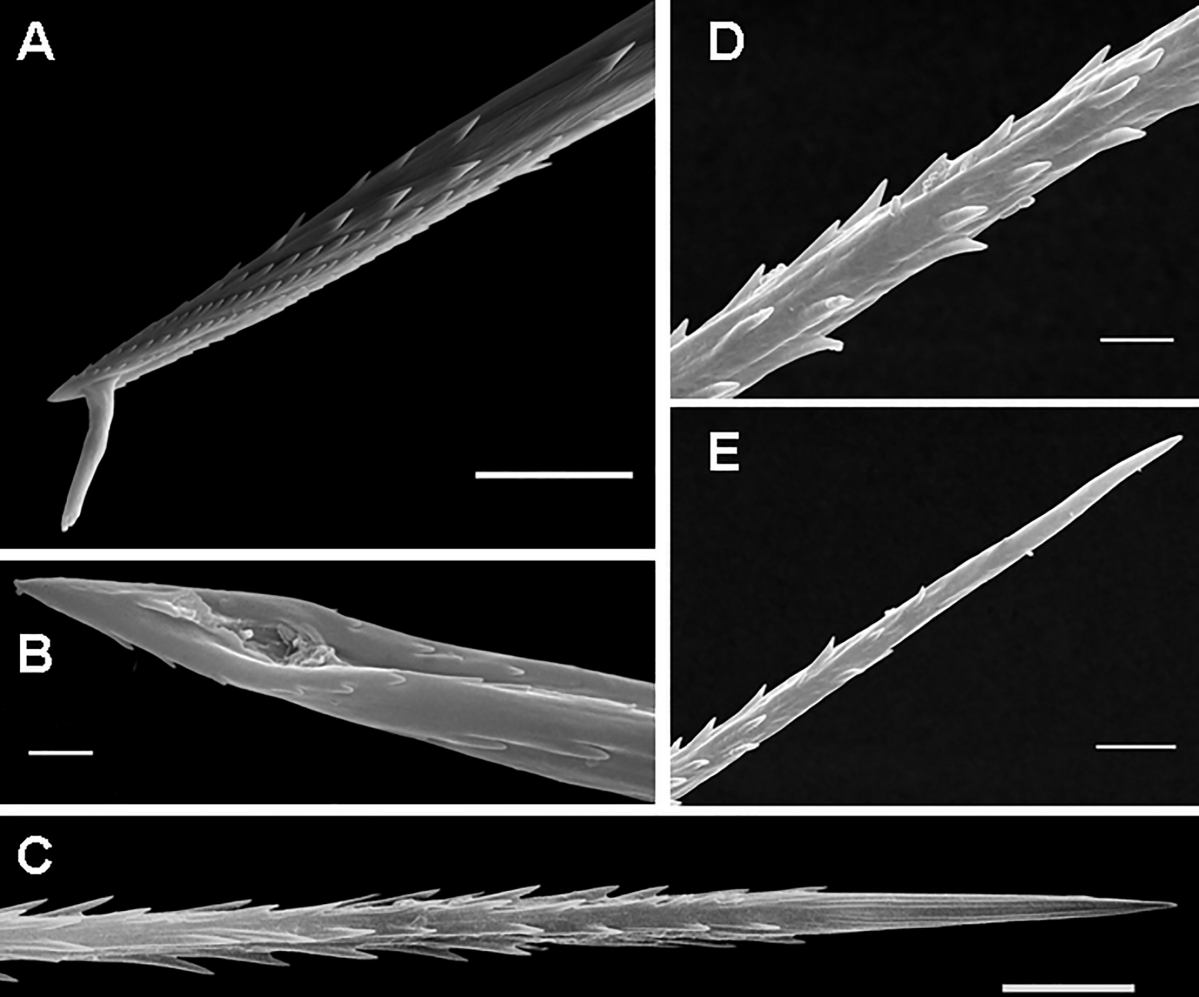
Urticating setae of type II. (A) *Avicularia* sp., female from Beni province, Bolivia. Type II, basal section with a supporting stalk. Scale bar = 20 μm. (B) *Caribena versicolor*, juv. (car. 3.5). Type II, basal section. Scale bar = 2 μm. (C) *Caribena versicolor*, male. Type II, apical section. Scale bar = 10 μm. (D) *Caribena versicolor*, juv. (car. 2.5). Type II, midsection. Basal part on the left. Scale bar = 5 μm. (E) *Caribena versicolor*, juv. (car. 3.5). Type II, apical section. The basic morphology of urticating setae in juveniles is identical to mature specimens, but the barbs are less developed. Scale bar = 10 μm. Abbreviations: juv. = juvenile specimen; car. = length of carapace.

**Fig 8:**
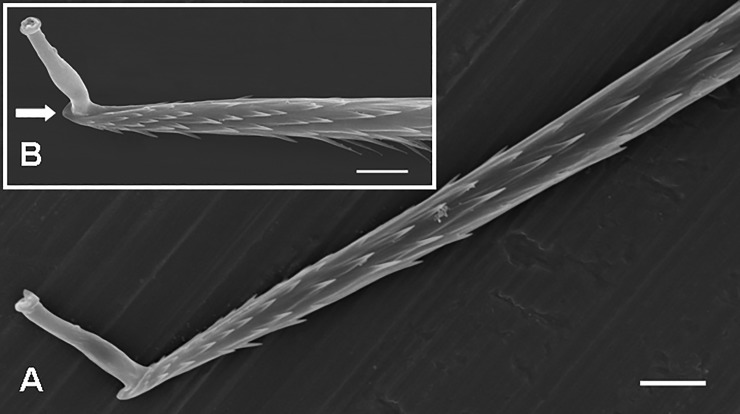
Urticating setae of type II in a female of *Antillena rickwesti*. (A) Type II, basal section with a supporting stalk, the apical section is bare. (B) Seta of intermediate morphology between body setae and type II, basal section with a supporting stalk. The arrow shows a precursor of the basal tip. Scale bar = 10 μm.

**Fig 9:**
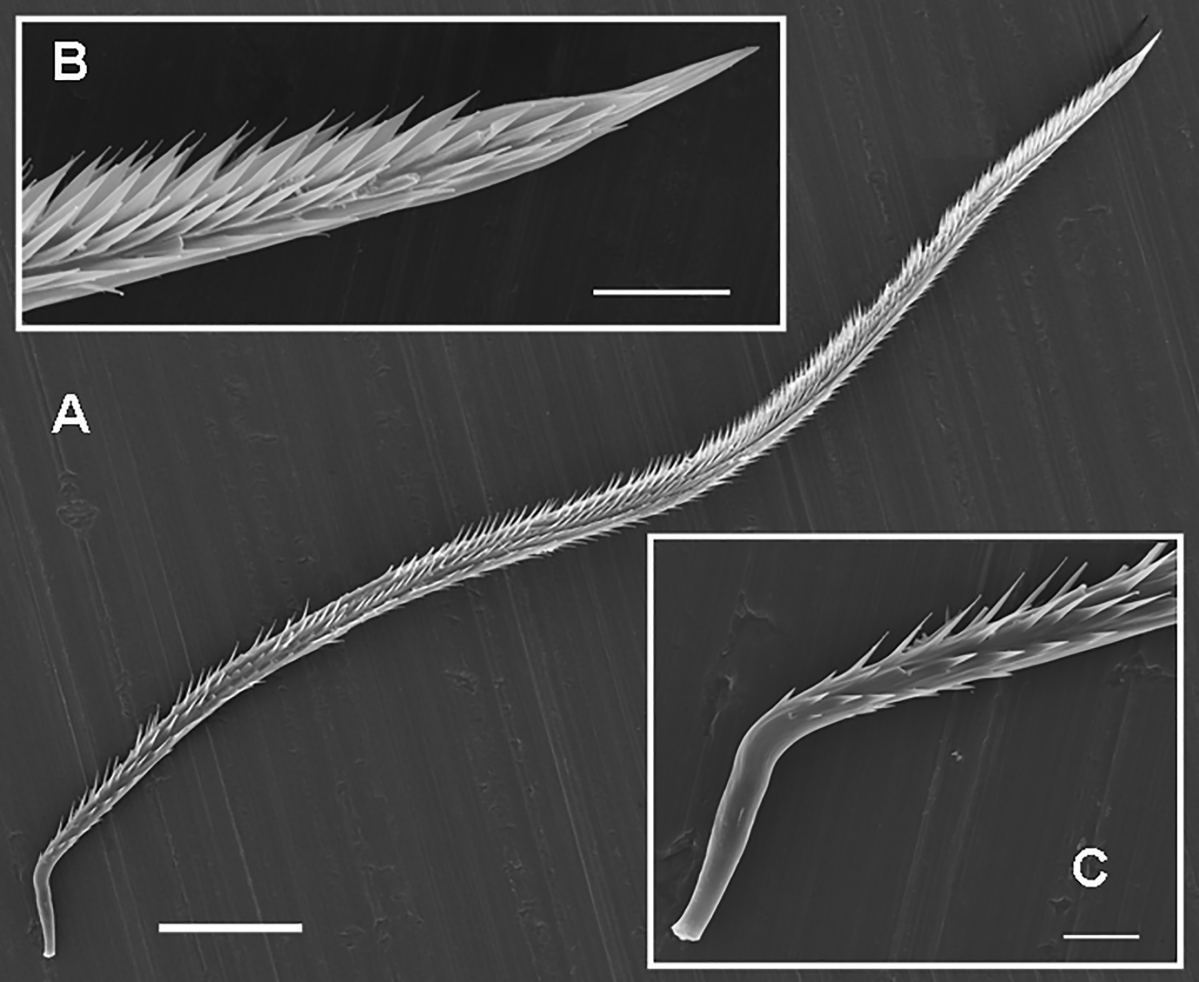
Urticating setae of type II and body seta of an immature female of *Iridopelma hirsutum*. (A) Type II, detail of the midsection. Scale bar = 5 μm. (B) Body seta, detail of the midsection. Scale bar = 5 μm. (C) Type II, detail of the basal section with a supporting stalk. Scale bar = 10 μm.

**Type II UrS** (Figs 1, 7A–7E, 8 and 9; Table 9): it is characterised by a stout and almost straight shaft with a basal and apical penetrating tip and with two axial sections (from the basal to the apical end): the basal section is equipped with short barbs or scale-like barbs (Fig 9C), which are restricted to the basal third only, with the exception of the UrS of *Caribena versicolor* (Walckenaer, 1837) and C. *laeta* (C. L. Koch, 1842) [14], whose barbs are longer and scattered along the whole shaft except the apex. The tapering apical section is bare. The morphology of UrS in two studied nymphs of *C*. *versicolor* (length of carapace (car.) 2.5 and 3.5) (Fig 7B, 7D and 7E) is congruent with that of the adults. The basal tips of the tested Aviculariinae were slightly curved upwards.

**Table 9:** Length ranges of type II setae, body setae and setae of intermediate morphology. juv. = juvenile specimen. car. = length of carapace.

**Species**	**Sex**	**Length of UrS**
*Antillena rickwesti*	♀	0.49–0.50
*Avicularia* sp. from Venezuela	♂	0.72–0.87
*Avicularia* sp. from Peru, Puerto Maldonado	♀	0.45–0.51
*Caribena versicolor* (car. 2.5)	juv.	0.28
*Caribena versicolor* (car. 3.5)	juv.	0.49–0.52
*Caribena versicolor*	♂	1.47–1.66
*Iridopelma hirsutum* (car. 6.5)	juv.	0.37
**Species**	**Sex**	**Length of intermediate setae**
*Antillena rickwesti*	♀	0.48–0.51
*Iridopelma hirsutum* (car. 6.5)	juv.	0.33–0.42
**Species**	**Sex**	**Length of body setae**
*Antillena rickwesti*	♀	0.47–0.53
*Iridopelma hirsutum* (car. 6.5)	juv.	0.29–0.43

Type II urticating setae are typical for some Aviculariinae genera such as *Antillena* Fukushima & Bertani, 2017, *Avicularia* Lamarck, 1818, *Caribena* Fukushima & Bertani, 2017, *Iridopelma* Pocock, 1901, *Pachistopelma* Pocock, 1901, *Typhochlaena* C. L. Koch, 1850, and *Ybyrapora* Fukushima & Bertani, 2017 [14,15].

In most of Aviculariinae with type II setae, the UrS can be dispersed through direct contact with the intruder [6,7], or may be airborne (recorded in *C*. *versicolor*; [8]). The shafts of airborne setae carrying well-developed barbs are much longer and narrower than those of the setae released by direct contact [8]. The length/width ratio is approximately three times higher for airborne setae than for setae released by direct contact [8]. Bertani et al. [8] supposed that the airborne setae represent a homoplastic character shared with Theraphosinae and are a derived character for the Aviculariinae genera *Avicularia*, *Iridopelma* and *Pachistopelma*. According to Cooke et al. [6] and Bertani & Marques [7], the penetrating tip is located basally near the supporting stalk; the mechanism of release of type II UrS was described and determined by the latter authors. In the present study we observed that type II setae also penetrated the target through their apical tips in two Aviculariinae (females of *Avicularia* sp. from Bolivia and *Pachistopelma bromelicola* Bertani, 2012). Both females were stimulated and irritated by the oval piece of polystyrene hold in tweezers. The distribution area of type II setae covers the northern part of South America, including some Caribbean islands (Kaderka [57]: Fig 1).

**Fig 10:**
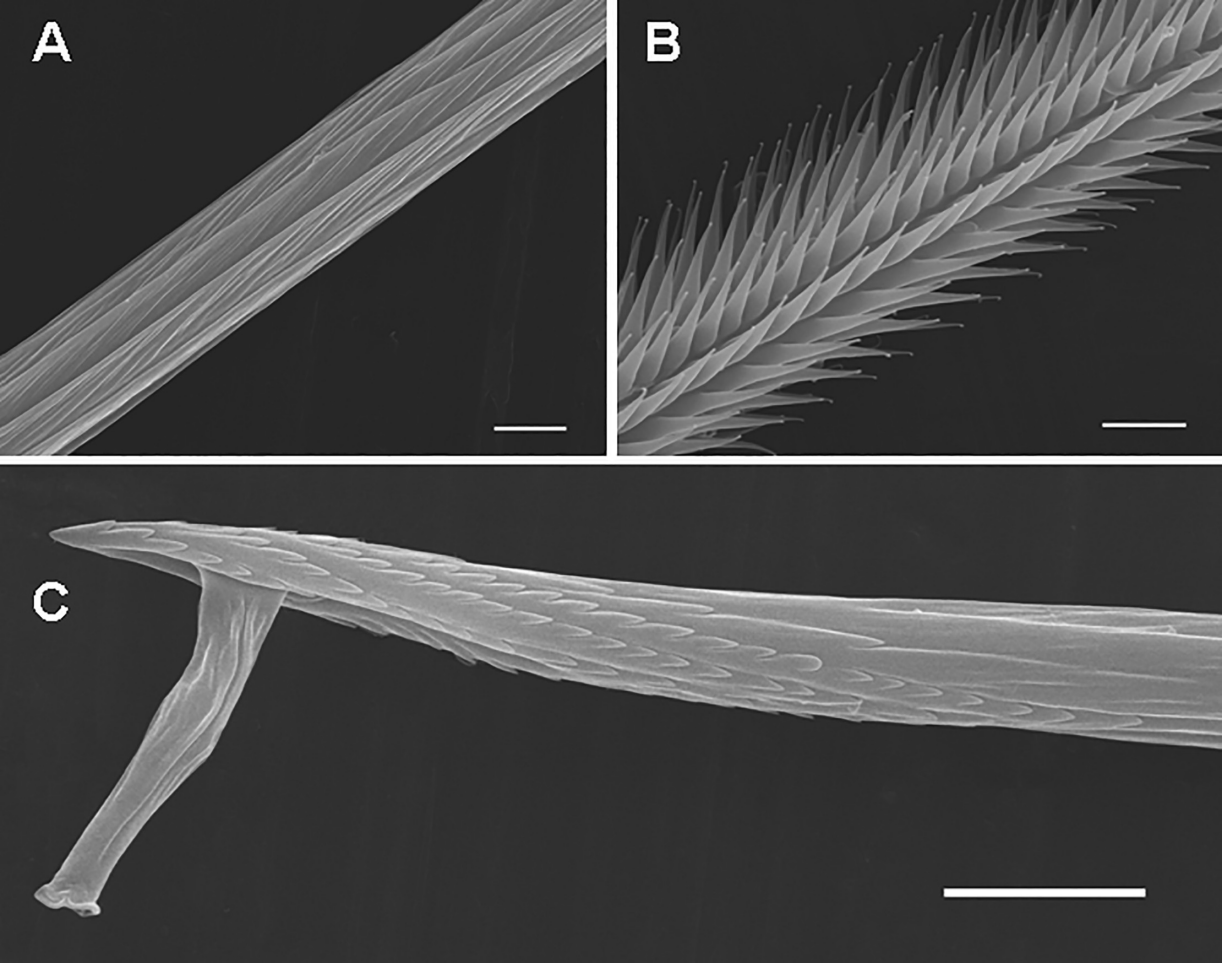
Body setae of a female of *Antillena rickwesti*. (A) Body seta. Scale bar = 50 μm. (B) Detail of the apical section. Scale bar = 10 μm. (C) Detail of the basal section with a supporting stalk. Scale bar = 10 μm.

The setae of intermediate morphology between the body setae and the type II urticating setae (Fig 8B), which were found in *Iridopelma hirsutum* Pocock, 1901 and *Antillena rickwesti* (Bertani & Huff, 2013), provide another evidence that type II UrS evolved from the body setae (Figs 9B and 10). These setae have a scale morphology restricted to the basal end only and are up to one fifth of the seta length. The apical section is densely covered with short barbs arranged in many longitudinal rows. In addition, they are nearly the same length as the body setae. Length of UrS: 0.45–1.66. Length of setae of intermediate morphology: 0.48–0.51. Length of body setae: 0.47–0.53.

**Fig 11:**
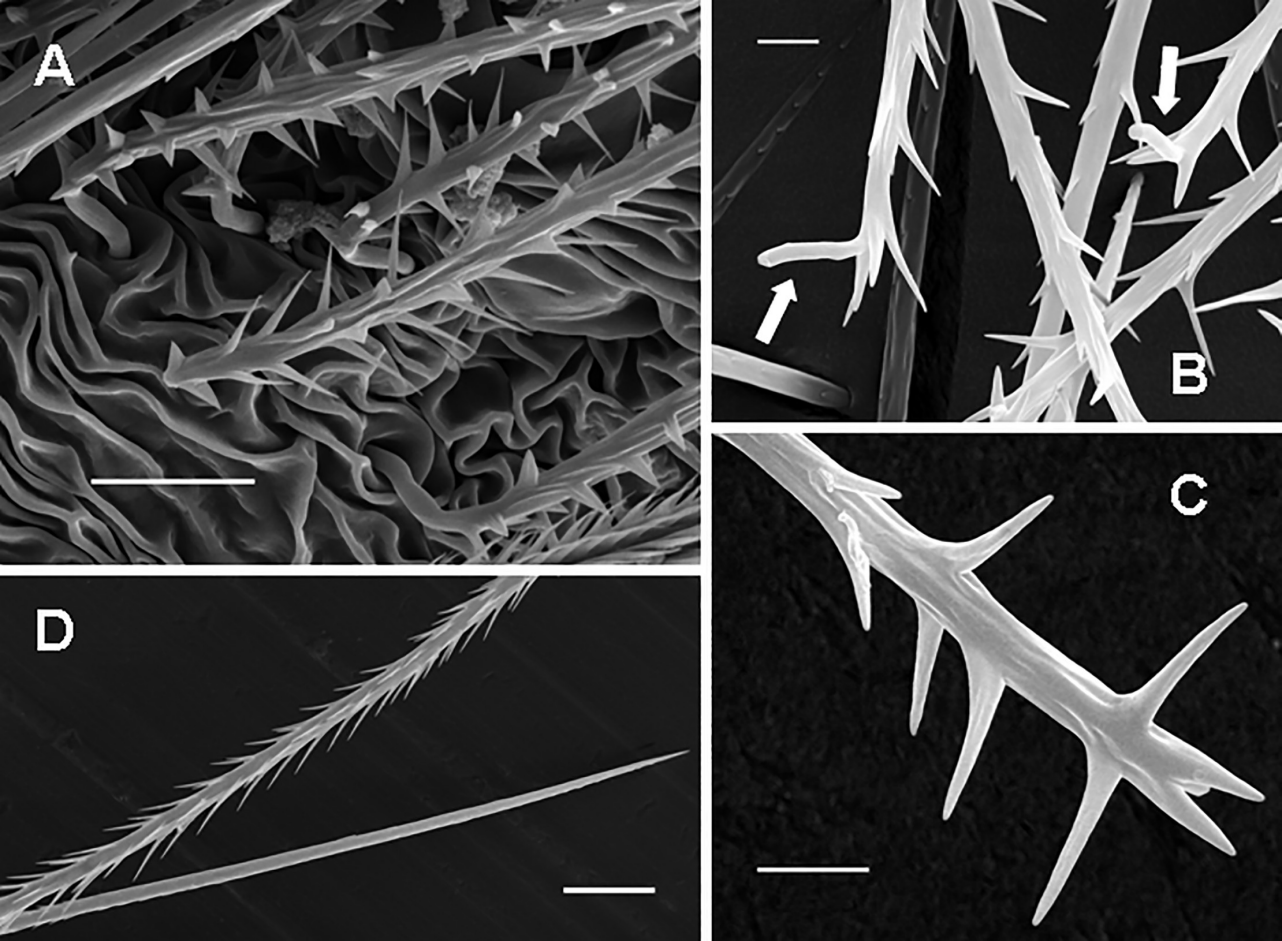
Urticating setae of type III. (A) *Cyriocosmus perezmilesi*, juv., a stage of the first nymph (according to Foelix [56]). Ontogenetic precursor of type III urticating setae, detail of the basal sections with non-reversed barbs. The arrangement of barbs in this stage is congruent with that of body setae presented by Bertani & Guadanucci [9]: Fig 25. Scale bar = 10 μm. (B) *Hapalopus* sp., juv. (car. 1.8), Lara State, Venezuela. Type III, supporting stalks in the basal sections marked by arrows. Scale bar = 5 μm. (C) *Hapalopus* sp., juv. (car. 1.8), Lara State, Venezuela. Type III urticating seta with unusually developed basal barbs, detail of the basal section. Scale bar = 5 μm. (D) *Hapalotremus* sp., a female from Peru. Type III, the apical (lower urticating seta) and the basal section (upper urticating seta). Scale bar = 20 μm. Abbreviations: juv. = juvenile specimen; car. = length of carapace.

**Fig 12:**
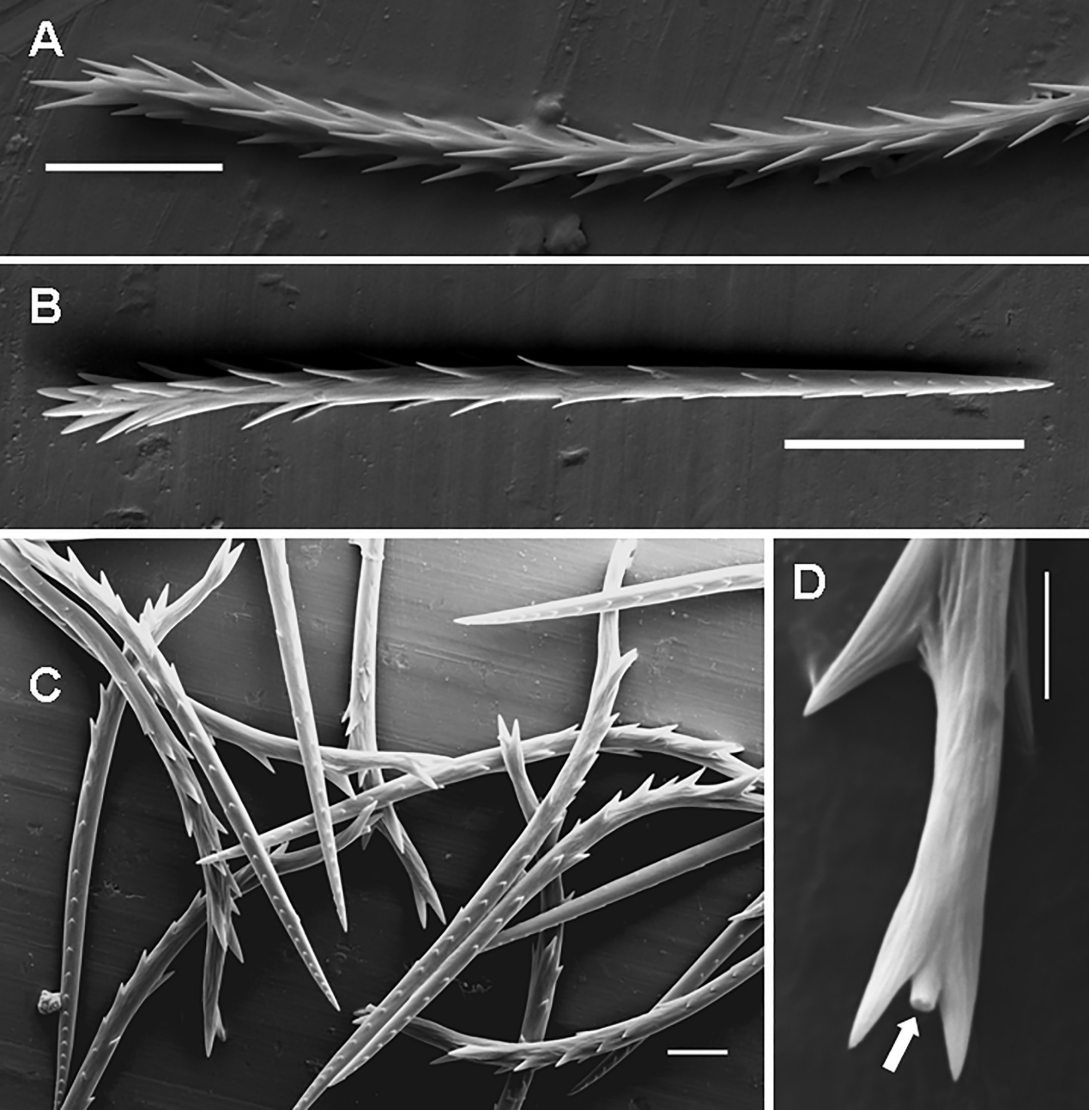
Urticating setae of type III and IV. (A) *Theraphosa blondi*, female. Long seta of type III, basal section, total length 0.28–0.32. Scale bar = 20 μm. (B) *Theraphosa blondi*, female. Short seta of type III, total length 0.07–0.08. Scale bar = 20 μm. (C) *Chromatopelma cyanopubescens*, female. Type IV with reversed barbs in the basal section and reversed denticles in the apical section. Scale bar = 10 μm. (D) *Chromatopelma cyanopubescens*, female. Type IV, basal end, the arrow shows the connection to a supporting stalk, located between the last two basal barbs. Scale bar = 5 μm.

**Type III UrS** (Figs 1, 11A–11D, 12A and 12B; Table 10): the seta is characterised by an almost straight shaft and by two axial sections: the long basal section has reversed barbs that are usually arranged in 4–5 longitudinal rows, the basal end of the shaft is slightly tapered. The connection of the seta with a supporting stalk is between the tips and basal ends of the basalmost reversed barbs. The short, tapering apical section lacks barbs but has reversed denticles. Length of setae: 0.07–1.25. Length of reversed barbs: 0.003–0.013. Setae of this type are usually arranged in one dorsal patch (most Theraphosinae) or in two dorsolateral patches, e.g., in *Phrixotrichus*, *Bistriopelma*, *Magulla* [39], and *Tmesiphantes hypogeus* Bertani, Bichuette & Pedroso, 2013 [58].

**Table 10:** Length ranges of type III setae. juv. = juvenile specimen. car. = length of carapace.

Species	Sex	Length of setae
*Bonnetina rudloffi*	♀	0.43–0.49
*Bonnetina* sp. from Mexico	♂	0.37–0.47
*Chromatopelma cyanopubescens*	♂	0.36–0.41
*Cyriocosmus perezmilesi*	♂	0.30–0.36
*Cyriocosmus perezmilesi*	♀	0.20–0.27
*Cyriocosmus venezuelensis*	♂	0.20–0.24
*Cyriocosmus venezuelensis*	♀	0.20–0.27
*Davus* sp. (1)	♂	0.36–0.41
*Davus pentaloris* from Mexico	♀	0.34–0.48
*Davus ruficeps*	♂	0.39–0.48
*Euathlus* sp. from Chile, Volcán Chilán	♀	0.35–0.46
*Grammostola* sp. from Argentina	♀	0.33–0.47
*Grammostola* sp. from Chile	♂	0.37–0.49
*Hapalotremus* sp. from Peru	♀	0.96–1.25
*Phrixotrichus vulpinus*	♂	0.47–0.53
*Phrixotrichus vulpinus*	♀	0.49–0.57
*Phrixotrichus vulpinus* (car. 2.2)	juv.	0.23–0.24
*Schizopelma* sp. from Mexico	♂	0.32–0.38
*Theraphosa blondi*	♀	short 0.07–0.08
*Theraphosa blondi*	♀	long 0.28–0.32
*Thrixopelma ockerti*	♀	0.44–0.61
*Tmesiphantes hypogeus*	♀	0.32

**Type IV UrS** (Figs 1, 12C and 12D; Table 11): the seta is characterised by a small size (up to 0.20) and a bent shaft. The basal section has strong reversed barbs (the diameter of the barbs at the basal end is comparable to the diameter of the shaft at the connection site). The central section has small reversed barbs arranged asymmetrically along the shaft. The tapering apical section has two opposite rows of reversed denticles. The connection of the seta with a supporting stalk is between the tips and basal ends of the well-developed basalmost reversed barbs. Length of setae: 0.08–0.21. Length of reversed barbs: 0.004–0.009. According to Bertani & Guadanucci [9], type IV UrS can be better characterised by the barbs pointing towards the convex side of the seta. The occurrence of this type is restricted to the South American Theraphosinae only (see Table 1).

**Table 11:** Length ranges of type IV setae.

Species	Sex	Length of setae
*Chromatopelma cyanopubescens*	♀	0.10–0.12
*Euathlus* sp. from Chile, Volcán Chillán	♀	0.11–0.13
*Grammostola* sp. from Argentina	♀	0.13–0.21
*Grammostola* sp. from Chile	♂	0.08–0.15
*Phrixotrichus vulpinus*	♀	0.15–0.19
*Thrixopelma ockerti*	♀	0.10–0.14

**Fig 13:**
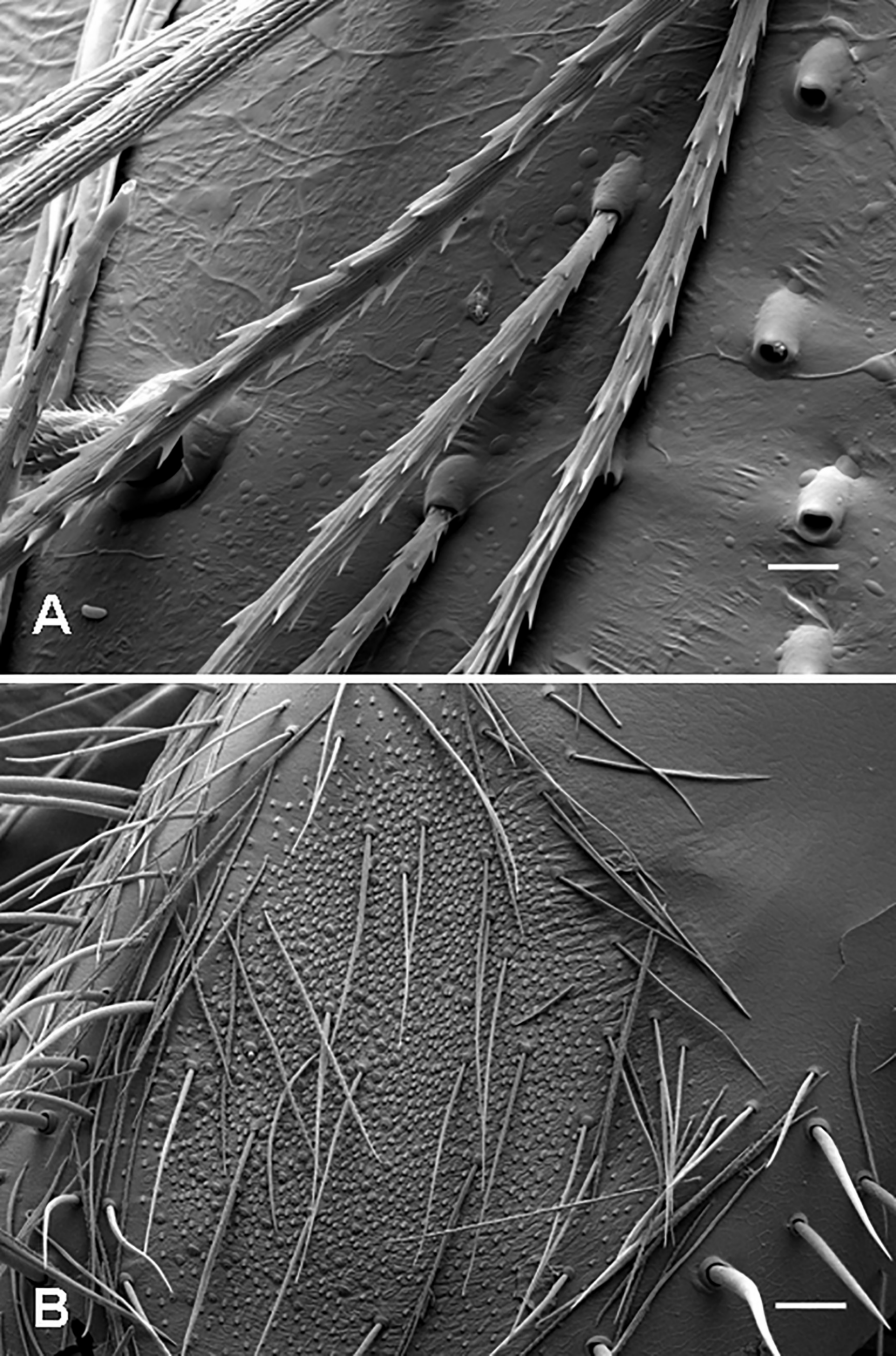
Palpal urticating setae of type V in an immature female of *Ephebopus cyanognathus*. (A) Type V urticating setae connected with the palpal surface by insertion sockets. Scale bar = 10 μm. (B) Prolateral face of the right palp with a limited area of insertion sockets without urticating setae. Scale bar = 100 μm.

**Type V UrS** (Figs 1, 13A and 13B; Table 12): the seta is characterised by an almost straight shaft and by two axial sections. The long basal section (80–90% of the seta length) has barbs arranged asymmetrically along the shaft; the angle between the shaft and the barbs is approximately 10–30° and the barbs are more confluent on the base. The short, tapering apical section is bare. Type V setae are inserted in the sockets on the palpal cuticle, and supporting stalks are absent. The seta break-off zone is inside the socket. Length of setae: 0.55–0.67. Length of barbs: 0.009–0.011. This type of urticating setae is located distally on the prolateral face of the palpal femora of *Ephebopus* spp. [59,60]. The setae are densely packed and uniformly arranged (Fig 13). This unique morphological characteristic is considered a generic characteristic of *Ephebopus* [59]. According to Bertani & Marques [7], there is no reason to consider this type of setae as homologous to the abdominal setae in Aviculariinae or Theraphosinae.

**Table 12:** Length ranges of type V setae. juv. = juvenile specimen.

Species	Sex	Length of setae
*Ephebopus cyanognathus*	juv.	0.60–0.67
*Ephebopus rufescens*	♀	0.55–0.62

**Fig 14:**
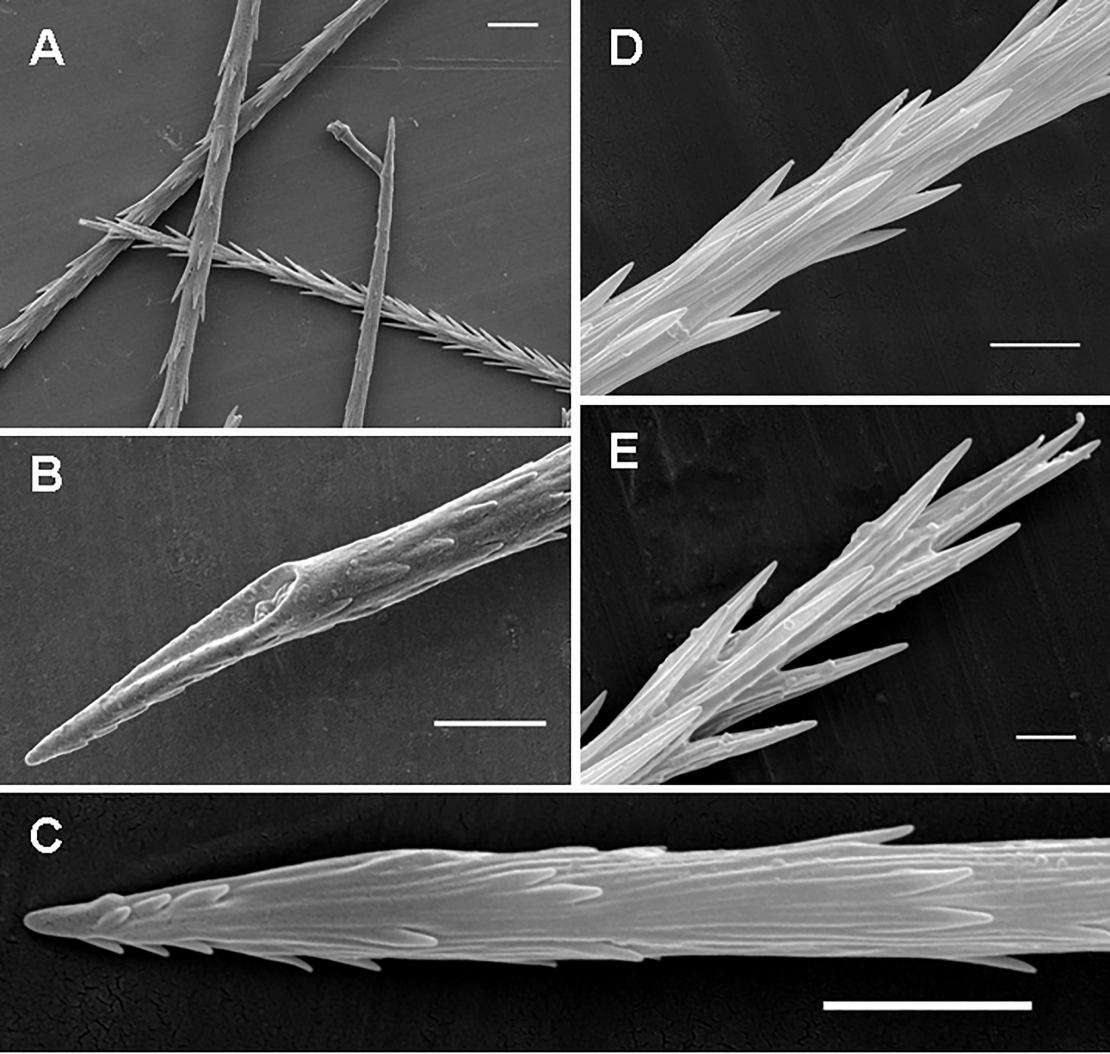
Urticating setae of type VI. **(**A) *Hemirrhagus papalotl*, female. Type VI, two midsections with short, more confluent barbs, apical section with longer and more protruding barbs, basal section with a supporting stalk. Scale bar = 10 μm. (B) *Hemirrhagus eros*, female. Type VI, the basal section with a socket for supporting stalk. Scale bar = 5 μm. (C) *Hemirrhagus papalotl*, immature male. Type VI, detail of the basal section. Scale bar = 5 μm. (D) *Hemirrhagus papalotl*, immature male. Type VI, detail of the midsection. Scale bar = 5 μm. (E) *Hemirrhagus papalotl*, immature male. Type VI, detail of the apical section. Scale bar = 2 μm.

**Fig 15:**
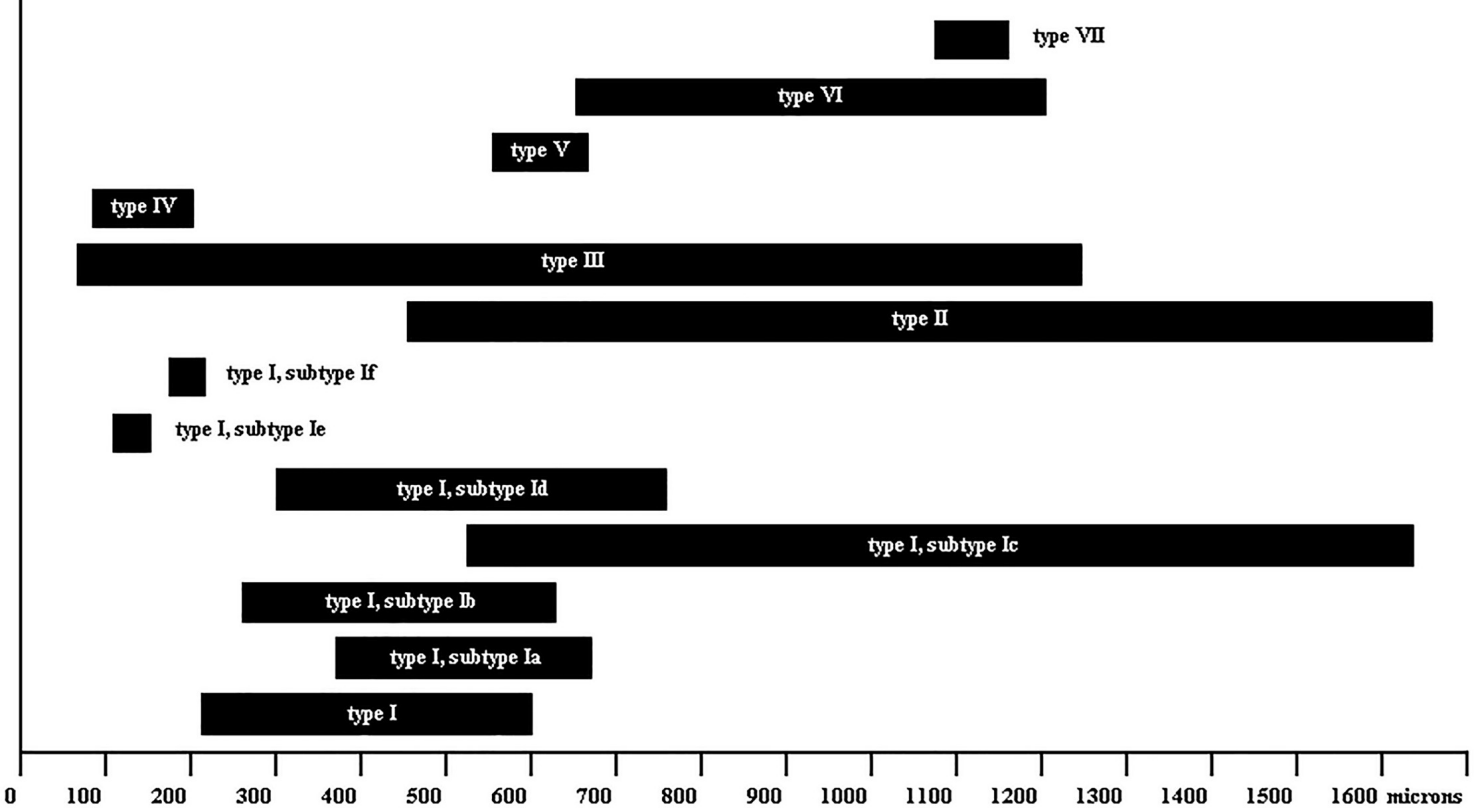
Length ranges of urticating setae types and their subtypes.

**Type VI UrS** (Figs 1, 14A–14E and 15; Pérez-Miles [13]: Figs 1–4; Table 13): this seta, with an almost straight shaft, is connected to the abdominal surface by cylindrical supporting stalks (Fig 14A). The barbs are subbasally short and more confluent to the shaft (for approximately 10% of the seta length) and longer and more protruding on the rest of the seta (the angle between the shaft and the barbs is approximately 30°). The apical section (approximately 10% of the seta length) can be bare (Pérez-Miles [13]: Figs 1–4) or can have well-developed protruding barbs reaching the apex, and causing the absence of the apical tip. Reversed barbs are absent on type VI setae. Length of setae: 0.64–1.21. Length of barbs (measured in apical region): 0.007–0.010. The setae can be arranged in one dorsomedial patch, two dorsal paramedian patches, or in two lateral patches [35]. This type was found only in the Mexican genus *Hemirrhagus*, with the exception of some troglobitic species [35]. Type VI setae are distinguished from the very similar type II setae by the presence of long barbs in the apical half. The morphology of the basal half is congruent.

**Table 13:** Length ranges of type VI setae. juv. = juvenile specimen. car. = length of carapace.

Species	Sex	Length of setae
*Hemirrhagus coztic*	♀	0.82–0.88
*Hemirrhagus eros*	♀	1.13–1.21
*Hemirrhagus papalotl*	♀	0.74–1.04
*Hemirrhagus papalotl* (car. 9.0)	juv. ♂	0.64–0.80

**Type VII UrS** (Perafán et al. [11]: Figs 2 and 8): the seta is straight and has small reversed subtriangular barbs or denticles along the entire shaft. These barbs are not homogenous in size or density and they are longer in the basal part. A small oval patch of lanceolated barbs is located in the apical quarter near the penetrating tip. The lanceolated barbs are longer, broader and less acute than the reversed barbs. The length/width ratio of the shaft is approximately 34:1. The setae are connected to the abdominal surface by thinner stalks. Type VII seta resembles type II but differs in the presence of the patch of lanceolated barbs and in the presence of reversed barbs scattered along the shaft [11]. These setae were found exclusively in *Kankuamo* [11].

The urticating setae types found at the examined specimens are listed in Table 14.

**Table 14:** Distribution of urticating setae types in studied material.

			URTICATING SETAE TYPES											
Species	Locality	Sex, stage	I	I_a_	I_b_	I_c_	I_d_	I_e_	I_f_	II	III	IV	V	VI
ISCHNOCOLINAE														
*Holothele* sp.	Peru, Rio Napo	♂	–	–	–	–	–	–	–	–	–	–	–	–
*Holothele* sp.	Venezuela, Aragua	♂	–	–	–	–	–	–	–	–	–	–	–	–
*Holothele* sp.	Venezuela, State of Bolívar	♂	–	–	–	–	–	–	–	–	–	–	–	–
*Holothele* sp.	Venezuela, State of Bolívar	♀	–	–	–	–	–	–	–	–	–	–	–	–
*Holothele* sp.	Venezuela, State of Carabobo	♂	–	–	–	–	–	–	–	–	–	–	–	–
SCHISMATOTHELINAE														
*Euthycaelus colonicus*	Venezuela, State of Carabobo	♂	–	–	–	–	–	–	–	–	–	–	–	–
*Neoholothele* sp.	Colombia	♀	–	–	–	–	–	–	–	–	–	–	–	–
*Neoholothele* sp.	Venezuela, Isla Margarita	♀	–	–	–	–	–	–	–	–	–	–	–	–
*Neoholothele* sp.	Venezuela, Isla Margarita	♂	–	–	–	–	–	–	–	–	–	–	–	–
*Schismatothele* sp.	Venezuela, State of Bolívar	♂	–	–	–	–	–	–	–	–	–	–	–	–
*Schismatothele* sp.	Venezuela, State of Mérida	♂	–	–	–	–	–	–	–	–	–	–	–	–
*Schismatothele* sp.	Venezuela, State of Guarico	♂	–	–	–	–	–	–	–	–	–	–	–	–
*Schismatothele* sp.	Venezuela, State of Guarico	♀	–	–	–	–	–	–	–	–	–	–	–	–
*Schismatothele* sp.	Venezuela, State of Aragua, Maracay	♂	–	–	–	–	–	–	–	–	–	–	–	–
PSALMOPOEINAE														
*Ephebopus cyanognathus*	French Guyana	juv. (car. 6.0)	–	–	–	–	–	–	–	–	–	–	+	–
*Ephebopus rufescens*	French Guyana	♀	–	–	–	–	–	–	–	–	–	–	+	–
*Psalmopoeus cambridgei*	unknown origin	♂	–	–	–	–	–	–	–	–	–	–	–	–
*Psalmopoeus irminia*	unknown origin	♀	–	–	–	–	–	–	–	–	–	–	–	–
*Psalmopoeus reduncus*	unknown origin	♂	–	–	–	–	–	–	–	–	–	–	–	–
*Tapinauchenius* sp.	Colombia, Puerto Arica	juv. ♀ (car. 2.2)	–	–	–	–	–	–	–	–	–	–	–	–
*Tapinauchenius* sp.	Colombia, Puerto Arica	♀	–	–	–	–	–	–	–	–	–	–	–	–
*Tapinauchenius* sp.	Venezuela, State of Delta Amacuro	♀	–	–	–	–	–	–	–	–	–	–	–	–
*Tapinauchenius* sp.	Peru	♂	–	–	–	–	–	–	–	–	–	–	–	–
AVICULARIINAE														
*Antillena rickwesti*	Dominican Republic	♀	–	–	–	–	–	–	–	+	–	–	–	–
*Avicularia hirschii*	Ecuador	♀	–	–	–	–	–	–	–	+	–	–	–	–
*Avicularia hirschii*	Ecuador	juv. (car. 3.0)	–	–	–	–	–	–	–	+	–	–	–	–
*Avicularia* sp.	Bolivia, Beni province	♀	–	–	–	–	–	–	–	+	–	–	–	–
*Avicularia* sp.	Peru, Puerto Maldonado	♀	–	–	–	–	–	–	–	+	–	–	–	–
*Avicularia* sp.	Venezuela	♂	–	–	–	–	–	–	–	+	–	–	–	–
*Caribena versicolor* (1)	unknown origin	♂	–	–	–	–	–	–	–	+	–	–	–	–
*Caribena versicolor* (2)	unknown origin	juv. (car. 2.5)	–	–	–	–	–	–	–	+	–	–	–	–
*Caribena versicolor* (3)	unknown origin	juv. (car. 3.5)	–	–	–	–	–	–	–	+	–	–	–	–
*Iridopelma hirsutum*	Brazil, Pernambuco, Recife	juv. ♀ (car. 6.5)	–	–	–	–	–	–	–	+	–	–	–	–
*Pachistopelma bromelicola*	Brazil	♀	–	–	–	–	–	–	–	+	–	–	–	–
THERAPHOSINAE														
*Acanthoscurria geniculata*	unknown origin	juv. ♀ (car. 1.7)	+	–	–	–	–	–	–	–	–	–	–	–
*Acanthoscurria geniculata*	unknown origin	juv. ♀ (car. 6.0)	+	+	+	+	–	–	–	–	–	–	–	–
*Acanthoscurria geniculata*	unknown origin	juv. ♀ (car. 7.3)	+	–	–	+	–	–	–	–	–	–	–	–
*Acanthoscurria geniculata*	unknown origin	♀	+	–	–	+	–	–	–	–	–	–	–	–
*Acanthoscurria* sp.	Paraguay, Terr. Fonciére	♀	+	–	–	+	–	–	–	–	–	–	–	–
*Acanthoscurria* sp.	Peru, Puerto Maldonado	♀	+	–	–	–	–	–	–	–	–	–	–	–
*Acanthoscurria* sp.	Peru, Puerto Maldonado	juv. (car. 1.7)	–	–	–	–	+	–	–	–	–	–	–	–
*Acanthoscurria suina*	Uruguay	♂	+	–	–	+	–	–	–	–	–	–	–	–
*Acanthoscurria theraphosoides*	Brazil	♂	+	–	–	+	–	–	–	–	–	–	–	–
*Aenigmarachne sinapophysis* ^a^	Costa Rica	♂	–	–	+	–	–	–	–	–	–	–	–	–
*Agnostopelma gardel*	Colombia, Boyacá, Belen	juv. (car. 2.8)	–	–	–	–	–	–	–	–	–	+	–	–
*Aphonopelma bicoloratum* (1)	Mexico	juv. ♂ (car. 7.0)	+	–	–	–	–	–	–	–	–	–	–	–
*Aphonopelma bicoloratum* (1)	Mexico	juv. ♂ (car. 10.0)	+	–	–	–	–	–	–	–	–	–	–	–
*Aphonopelma bicoloratum* (1)	Mexico	juv. ♂ (car. 14.5)	+	–	–	–	–	–	–	–	–	–	–	–
*Aphonopelma bicoloratum* (2)	Mexico	♂ (car. 15.0)	+	+	+	–	–	–	–	–	–	–	–	–
*Aphonopelma crinirufum*	Costa Rica, Puntarenas	♀	+	–	–	–	–	–	–	–	–	–	–	–
*Aphonopelma crinirufum*	Costa Rica, Peninsula de Nicoya	♂	+	+	–	–	–	–	–	–	–	–	–	–
*Aphonopelma seemanni*	Costa Rica	♀	+	–	–	–	–	–	–	–	–	–	–	–
*Aphonopelma seemanni*	Costa Rica, Alajuela province	♂	–	+	+	–	–	–	–	–	–	–	–	–
*Bistriopelma lamasi*	Peru, Ayacucho province, Pampa Galeras	♂	–	–	–	–	–	–	–	–	+	–	–	–
*Bistriopelma lamasi*	Peru, Ayacucho province, Pampa Galeras	♀	–	–	–	–	–	–	–	–	+	–	–	–
*Bistriopelma matuskai*	Peru, Apurímac province, Abancay	♂	–	–	–	–	–	–	–	–	+	–	–	–
*Bistriopelma matuskai*	Peru, Apurímac province, Abancay	♀	–	–	–	–	–	–	–	–	+	–	–	–
*Bistriopelma* sp.	Peru, Puno, Isla Amantani	♀	–	–	–	–	–	–	–	–	+	–	–	–
*Bonnetina rudloffi*	Mexico	♀	–	–	–	–	–	–	–	–	+	–	–	–
*Bonnetina* sp.	Mexico	♂	–	–	–	–	–	–	–	–	+	–	–	–
*Brachypelma albiceps*	unknown origin	♀	+	–	–	+	–	–	–	–	–	–	–	–
*Brachypelma albopilosum* (1)	unknown origin	juv. (car. 2.1)	+	–	–	–	–	–	–	–	–	–	–	–
*Brachypelma albopilosum* (2)	unknown origin	juv. ♀ (car. 14.0)	+	+	+	–	–	–	–	–	–	–	–	–
*Brachypelma auratum*	Mexico	♀	+	–	–	+	–	–	–	–	–	–	–	–
*Brachypelma baumgarteni*	Mexico	♀	+	–	–	+	–	–	–	–	–	–	–	–
*Brachypelma baumgarteni*	Mexico	♂	+	–	–	+	–	–	–	–	–	–	–	–
*Brachypelma fossorium*	Costa Rica, Guanacaste province	♂	+	+	+	–	–	–	–	–	–	–	–	–
*Brachypelma klaasi*	Mexico	♀	+	+	–	+	–	–	–	–	–	–	–	–
*Brachypelma klaasi*	Mexico	♂	+	+	+	+	–	–	–	–	–	–	–	–
*Brachypelma smithi*	unknown origin	juv. ♀ (car. 21.0)	+	+	+	+	–	–	–	–	–	–	–	–
*Brachypelma* sp.	Mexico, Oaxaca	♂	–	+	+	–	–	–	–	–	–	–	–	–
*Brachypelma verdezi*	Mexico	♀	+	–	+	–	–	–	–	–	–	–	–	–
*Brachypelma verdezi*	Mexico	♂	–	+	+	–	–	–	–	–	–	–	–	–
*Chromatopelma cyanopubescens* (1)	Venezuela	♀	–	–	–	–	–	–	–	–	+	+	–	–
*Chromatopelma cyanopubescens* (2)	Venezuela	♂	–	–	–	–	–	–	–	–	+	–	–	–
*Chromatopelma cyanopubescens* (3)	Venezuela	juv. ♀ (car. 3.0)	–	–	–	–	–	–	–	–	+	+	–	–
*Chromatopelma cyanopubescens* (3)	Venezuela	juv. ♀ (car. 4.1)	–	–	–	–	–	–	–	–	+	+	–	–
*Chromatopelma cyanopubescens* (3)	Venezuela	juv. ♀ (car. 5.0)	–	–	–	–	–	–	–	–	+	+	–	–
*Chromatopelma cyanopubescens* (3)	Venezuela	juv. ♀ (car. 5.9)	–	–	–	–	–	–	–	–	+	+	–	–
*Chromatopelma cyanopubescens* (3)	Venezuela	juv. ♀ (car. 7.7)	–	–	–	–	–	–	–	–	+	+	–	–
*Chromatopelma cyanopubescens* (3)	Venezuela	juv. ♀ (car. 9.5)	–	–	–	–	–	–	–	–	+	+	–	–
*Chromatopelma cyanopubescens* (3)	Venezuela	juv. ♀ (car. 10.6)	–	–	–	–	–	–	–	–	+	+	–	–
*Chromatopelma cyanopubescens* (3)	Venezuela	juv. ♀ (car. 12.4)	–	–	–	–	–	–	–	–	+	+	–	–
*Chromatopelma cyanopubescens* (3)	Venezuela	juv. ♀ (car. 14.0)	–	–	–	–	–	–	–	–	+	+	–	–
*Chromatopelma cyanopubescens* (3)	Venezuela	juv. ♀ (car. 17.3)	–	–	–	–	–	–	–	–	+	+	–	–
*Chromatopelma cyanopubescens* (3)	Venezuela	juv. ♀ (car. 18.8)	–	–	–	–	–	–	–	–	+	+	–	–
*Chromatopelma cyanopubescens* (4)	Venezuela	juv. ♂ (car. 3.0)	–	–	–	–	–	–	–	–	+	+	–	–
*Chromatopelma cyanopubescens* (4)	Venezuela	juv. ♂ (car. 4.0)	–	–	–	–	–	–	–	–	+	+	–	–
*Chromatopelma cyanopubescens* (4)	Venezuela	juv. ♂ (car. 5.0)	–	–	–	–	–	–	–	–	+	+	–	–
*Chromatopelma cyanopubescens* (4)	Venezuela	juv. ♂ (car. 6.0)	–	–	–	–	–	–	–	–	+	+	–	–
*Chromatopelma cyanopubescens* (4)	Venezuela	juv. ♂ (car. 7.5)	–	–	–	–	–	–	–	–	+	+	–	–
*Chromatopelma cyanopubescens* (4)	Venezuela	juv. ♂ (car. 9.2)	–	–	–	–	–	–	–	–	+	+	–	–
*Chromatopelma cyanopubescens* (4)	Venezuela	juv. ♂ (car. 10.5)	–	–	–	–	–	–	–	–	+	+	–	–
*Chromatopelma cyanopubescens* (4)	Venezuela	juv. ♂ (car. 13.2)	–	–	–	–	–	–	–	–	+	+	–	–
*Chromatopelma cyanopubescens* (4)	Venezuela	juv. ♂ (car. 15.0)	–	–	–	–	–	–	–	–	+	+	–	–
*Chromatopelma cyanopubescens* (4)	Venezuela	♂ (car. 16.5)	–	–	–	–	–	–	–	–	+	–	–	–
*Chromatopelma cyanopubescens* (5)	Venezuela	juv. (car. 4.5)	–	–	–	–	–	–	–	–	+	+	–	–
*Citharacanthus cyaneus*	Cuba, Granma	♂	+	+	+	–	+	–	–	–	–	–	–	–
*Citharacanthus livingstoni*	Guatemala, Livingston	♂	–	–	–	–	+	–	–	–	–	–	–	–
*Citharacanthus longipes*	Mexico, State of Chiapas	♀	–	–	–	–	+	–	–	–	–	–	–	–
*Citharacanthus longipes*	Mexico, State of Chiapas	♂	–	–	–	–	+	–	–	–	–	–	–	–
*Citharacanthus* sp.	Mexico, State of Veracruz	♂	+	+	–	–	+	–	–	–	–	–	–	–
Affinity to *Citharacanthus* ^b^ (1)	Costa Rica, Guapiles	♂	–	–	–	–	+	–	–	–	–	–	–	–
Affinity to *Citharacanthus* ^b^ (2)	Costa Rica, Guapiles	juv. ♀ (car. 2.1)	–	–	–	–	+	–	–	–	–	–	–	–
Affinity to *Citharacanthus* ^b^ (2)	Costa Rica, Guapiles	juv. ♀ (car. 10.5)	–	–	–	–	+	–	–	–	–	–	–	–
Affinity to *Citharacanthus* ^b^(2)	Costa Rica, Guapiles	♀	–	–	–	–	+	–	–	–	–	–	–	–
*Crassicrus lamanai*	unknown origin	♀	+	–	–	–	–	–	–	–	–	–	–	–
*Cyclosternum schmardae*	Ecuador, Cordillera	♀	+	+	–	–	–	–	–	–	–	–	–	–
*Cyclosternum* sp.	Peru, Puerto Maldonado	♀	+	–	–	–	–	–	–	–	–	–	–	–
*Cyclosternum* sp.	Peru, Ucayali, Pucallpa	♀	+	–	–	–	–	–	–	–	–	–	–	–
*Cyriocosmus leetzi*	Venezuela, State of Táchira	♂	–	–	–	–	–	–	–	–	+	–	–	–
*Cyriocosmus perezmilesi* (1)	Bolivia, Beni province	♂	–	–	–	–	–	–	–	–	+	–	–	–
*Cyriocosmus perezmilesi* (2)	Bolivia, Beni province	♀	–	–	–	–	–	–	–	–	+	–	–	–
*Cyriocosmus perezmilesi* (3)	Bolivia, Beni province	juv. (car. 1.5)	–	–	–	–	–	–	–	–	–	–	–	–
*Cyriocosmus perezmilesi* (3)	Bolivia, Beni province	juv. (car. 2.2)	–	–	–	–	–	–	–	–	+	–	–	–
*Cyriocosmus rogerioi*	Peru, Kuelap near Chachapoyas	♂	–	–	–	–	–	–	–	–	+	–	–	–
*Cyriocosmus venezuelensis*	Venezuela, State of Lara	♂	–	–	–	–	–	–	–	–	+	–	–	–
*Cyriocosmus venezuelensis*	Venezuela, State of Lara	♀	–	–	–	–	–	–	–	–	+	–	–	–
*Cyrtopholis flavostriatus* (1)	Guano, Lesser Antilles	♀	+	+	+	–	–	–	–	–	–	–	–	–
*Cyrtopholis flavostriatus* (2)	unknown origin	juv. ♀ (car. 7.5)	+	–	–	–	–	–	–	–	–	–	–	–
*Cyrtopholis flavostriatus* (2)	unknown origin	juv. ♀ (car. 18.0)	+	+	–	–	–	–	–	–	–	–	–	–
*Cyrtopholis* sp.	Cuba, Guantánamo	♂	+	+	+	–	–	–	–	–	–	–	–	–
*Cyrtopholis* sp.	Cuba, Holguín province	♂	+	+	+	–	–	–	–	–	–	–	–	–
*Cyrtopholis* sp. (1)	Cuba, Santiago de Cuba province, Baconao	juv. ♂	+	–	–	–	–	–	–	–	–	–	–	–
*Cyrtopholis* sp. (1)	Cuba, Santiago de Cuba province, Baconao	♂	–	+	+	–	–	–	–	–	–	–	–	–
*Cyrtopholis* sp. (2)	Cuba, Santiago de Cuba province, Baconao	♂	+	+	+	–	–	–	–	–	–	–	–	–
*Cyrtopholis* sp.	Cuba, Trinidad	♀	+	–	–	–	–	–	–	–	–	–	–	–
*Cyrtopholis* sp.	Dominican Republic, La Vega province	♀	+	–	–	–	–	–	–	–	–	–	–	–
*Cyrtopholis* sp. (1)	Dominican Republic, Pedernales province	♀	+	–	–	–	–	–	–	–	–	–	–	–
*Cyrtopholis* sp. (2)	Dominican Republic, Pedernales province	juv. (car. 2.7)	+	–	–	–	–	–	–	–	–	–	–	–
*Davus pentaloris*	Mexico, State of Oaxaca	♀												
*Davus ruficeps*	Costa Rica, Peninsula de Nicoya	♂	–	–	–	–	–	–	–	–	+	–	–	–
*Davus* sp. (1)	unknown origin	♂	–	–	–	–	–	–	–	–	+	–	–	–
*Davus* sp. (2)	unknown origin	juv. ♂ (car. 2.0)	–	–	–	–	–	–	–	–	+	–	–	–
*Davus* sp. (2)	unknown origin	juv. ♂ (car. 2.9)	–	–	–	–	–	–	–	–	+	–	–	–
*Davus* sp. (2)	unknown origin	juv. ♂ (car. 4.4)	–	–	–	–	–	–	–	–	+	–	–	–
*Davus* sp. (2)	unknown origin	juv. ♂ (car. 5.6)	–	–	–	–	–	–	–	–	+	–	–	–
*Davus* sp. (2)	unknown origin	juv. ♂ (car. 6.9)	–	–	–	–	–	–	–	–	+	–	–	–
*Davus* sp. (2)	unknown origin	juv. ♂ (car. 7.8)	–	–	–	–	–	–	–	–	+	–	–	–
*Davus* sp. (2)	unknown origin	juv. ♂ (car. 9.0)	–	–	–	–	–	–	–	–	+	–	–	–
*Davus* sp. (2)	unknown origin	juv. ♂ (car. 11.0)	–	–	–	–	–	–	–	–	+	–	–	–
*Davus* sp. (2)	unknown origin	juv. ♂ (car. 13.0)	–	–	–	–	–	–	–	–	+	–	–	–
*Davus* sp. (2)	unknown origin	♂ (car. 14.6)	–	–	–	–	–	–	–	–	+	–	–	–
*Euathlus* sp.	Chile	♂	–	–	–	–	–	–	–	–	+	+	–	–
*Euathlus* sp.	Chile, Volcán Chillán	♀	–	–	–	–	–	–	–	–	+	+	–	–
*Euathlus truculentus*	Chile, Santiago—Valparaiso	♂	–	–	–	–	–	–	–	–	+	+	–	–
*Eupalaestrus larae*	Argentina	♀	+	+	–	+	–	–	–	–	–	–	–	–
*Eupalaestrus weijenberghi* (1)	Uruguay	♂	+	–	+	+	–	–	–	–	–	–	–	–
*Eupalaestrus weijenberghi* (2)	Uruguay	♀	+	+	+	–	–	–	–	–	–	–	–	–
*Eupalaestrus weijenberghi* (3)	Uruguay	juv. ♀ (car. 4.2)	+	–	–	–	–	–	–	–	–	–	–	–
*Eupalaestrus weijenberghi* (3)	Uruguay	juv. ♀ (car. 6.8)	+	–	–	–	–	–	–	–	–	–	–	–
*Eupalaestrus weijenberghi* (3)	Uruguay	juv. ♀ (car. 10.2)	+	–	–	–	–	–	–	–	–	–	–	–
*Eupalaestrus weijenberghi* (3)	Uruguay	juv. ♀ (car. 11.7)	+	–	–	–	–	–	–	–	–	–	–	–
*Grammostola grossa*	Brazil	♂	–	–	–	–	–	–	–	–	+	–	–	–
*Grammostola pulchripes*	unknown origin	juv. ♀ (car. 12.0)	–	–	–	–	–	–	–	–	+	+	–	–
*Grammostola* sp. (1)	Chile	♂	–	–	–	–	–	–	–	–	+	+	–	–
*Grammostola* sp. (2)	Chile	juv. (car. 3.0)	–	–	–	–	–	–	–	–	–	+	–	–
*Grammostola* sp. (3)	Chile	juv. ♂ (car. 3.2)	–	–	–	–	–	–	–	–	–	+	–	–
*Grammostola* sp. (3)	Chile	juv. ♂ (car. 3.9)	–	–	–	–	–	–	–	–	–	+	–	–
*Grammostola* sp. (3)	Chile	juv. ♂ (car. 4.8)	–	–	–	–	–	–	–	–	–	+	–	–
*Grammostola* sp. (3)	Chile	juv. ♂ (car. 5.5)	–	–	–	–	–	–	–	–	–	+	–	–
*Grammostola* sp. (3)	Chile	juv. ♂ (car. 6.7)	–	–	–	–	–	–	–	–	–	+	–	–
*Grammostola* sp. (4)	Chile	♂	–	–	–	–	–	–	–	–	+	+	–	–
*Grammostola* sp.	Argentina	♀	–	–	–	–	–	–	–	–	+	+	–	–
*Hapalopus butantan*	Brazil, Amazonas	♀	–	–	–	–	–	–	–	–	–	+	–	–
*Hapalopus formosus*	Colombia, Bogotá	♂	–	–	–	–	–	–	–	–	+	–	–	–
*Hapalopus triseriatus*	Venezuela, State of Mérida	♀	–	–	–	–	–	–	–	–	+	–	–	–
*Hapalopus* sp.	Venezuela, State of Lara	♂	–	–	–	–	–	–	–	–	+	–	–	–
*Hapalopus* sp.	Venezuela, State of Lara	juv. (car. 1.8)	–	–	–	–	–	–	–	–	+	–	–	–
*Hapalopus* sp.	Costa Rica	♀	–	–	–	–	–	–	–	–	+	–	–	–
*Hapalopus* sp.	Costa Rica	♂	–	–	–	–	–	–	–	–	+	–	–	–
*Hapalotremus* sp.	Peru	♀	–	–	–	–	–	–	–	–	+	–	–	–
*Hapalotremus* sp.	Peru, Cusco, Tipon	♀	–	–	–	–	–	–	–	–	+	–	–	–
*Hapalotremus* sp.	Peru, Cusco, Ollantaytambo	♀	–	–	–	–	–	–	–	–	+	–	–	–
*Hapalotremus* sp.	Peru, Cusco, Calca, Pitusiray	♀	–	–	–	–	–	–	–	–	+	–	–	–
*Hapalotremus* sp.	Peru, Cusco, Calca, Pitusiray	♀ (car. 1.61)	–	–	–	–	–	–	–	–	–	–	–	–
*Hapalotremus* sp.	Peru, Cusco, Calca, Pitusiray	♀ (car. 1.64)	–	–	–	–	–	–	–	–	+	–	–	–
*Hemirrhagus coztic*	Mexico, Morelos, Tepoztlán, Cueva del Diablo	♀	–	–	–	–	–	–	–	–	–	–	–	+
*Hemirrhagus eros*	Mexico, Oaxaca, El Punto	♀	–	–	–	–	–	–	–	–	–	–	–	+
*Hemirrhagus ocellatus*	Mexico, Estado de Mexico, Cueva Peña Blanca	-	–	–	–	–	–	–	–	–	–	–	–	+
*Hemirrhagus papalotl*	Mexico, Guerrero, Gruta de Aguacachil, Taxco	♀	–	–	–	–	–	–	–	–	–	–	–	+
*Hemirrhagus papalotl*	Mexico, State of Guerrero, Cave La Joya	juv. ♂	–	–	–	–	–	–	–	–	–	–	–	+
*Homoeomma* sp.	Chile	♂	–	–	–	–	–	–	–	–	+	–	–	–
*Homoeomma* sp.	Chile	♀	–	–	–	–	–	–	–	–	+	+	–	–
*Kochiana brunnipes* (1)	unknown origin	♀	–	–	–	–	–	–	–	–	+	–	–	–
*Kochiana brunnipes* (2)	unknown origin	juv. (car. 1.0)	–	–	–	–	–	–	–	–	+	–	–	–
*Kochiana brunnipes* (2)	unknown origin	juv. ♀ (car. 3.1)	–	–	–	–	–	–	–	–	+	–	–	–
*Kochiana brunnipes* (2)	unknown origin	♀ (car. 10.3)	–	–	–	–	–	–	–	–	+	–	–	–
*Lasiodora isabelline*	Brazil, Rio de Janeiro	juv. ♀	+	–	+	+	–	–	–	–	–	–	–	–
*Magnacarina* sp.	Mexico, State of Oaxaca, Bahías de Huatulco	♂	–	–	–	–	–	–	–	–	+	–	–	–
*Megaphobema mesomelas*	unknown origin	♂	+	+	+	+	–	–	–	–	–	–	–	–
*Megaphobema robustum*	unknown origin	♀	+	–	–	+	–	–	–	–	–	–	–	–
*Megaphobema velvetosoma*	Ecuador	♀	+	–	–	+	–	–	–	–	–	–	–	–
*Metriopelma* sp. ^c^	Costa Rica, Alajuela province	♂	+	–	–	–	–	–	–	–	–	–	–	–
*Metriopelma* sp. ^c^	Costa Rica, Alajuela province	♀	+	–	–	–	–	–	–	–	–	–	–	–
*Metriopelma* sp. ^c^	Venezuela, Isla Margarita	♀	–	–	–	–	–	+	–	–	–	–	–	–
*Metriopelma* sp. ^c^	Venezuela, Isla Margarita	♂	–	–	+	–	–	–	–	–	–	–	–	–
*Metriopelma* sp. ^c^	Venezuela, State of Barinas	juv. (car. 7.5)	+	–	–	–	–	–	–	–	–	–	–	–
*Metriopelma* sp. ^c^	Venezuela, State of Aragua	juv. (car. 9.0)	+	–	–	–	–	–	–	–	–	–	–	–
*Metriopelma* sp. ^c^	Venezuela, State of Portuguese	♂	–	+	+	–	–	–	–	–	–	–	–	–
*Mygalarachne brevipes*	Honduras	♀	+	+	–	–	–	–	–	–	–	–	–	–
*Neischnocolus armihuariensis*	Peru, Cuzco province	♂	–	–	–	–	+	–	–	–	–	–	–	–
*Neischnocolus* sp.	Colombia, Ibaque Tolima	♂	–	–	+	–	+	–	–	–	–	–	–	–
*Neischnocolus* sp.	Costa Rica	♂	–	–	+	–	+	–	–	–	–	–	–	–
*Neischnocolus* sp.	Ecuador, Imbabura / Carchi province	♀	+	–	–	–	+	–	–	–	–	–	–	–
*Neischnocolus* sp.	Venezuela, State of Guarico	♀	+	–	–	–	+	–	–	–	–	–	–	–
*Nhandu coloratovillosus* (1)	unknown origin	♀	+	–	–	–	–	–	–	–	–	–	–	–
*Nhandu coloratovillosus* (2)	unknown origin	juv. ♂ (car. 3.7)	+	–	–	–	–	–	–	–	–	–	–	–
*Nhandu coloratovillosus* (2)	unknown origin	juv. ♂ (car. 6.0)	+	–	–	–	–	–	–	–	–	–	–	–
*Nhandu coloratovillosus* (2)	unknown origin	juv. ♂ (car. 7.4)	+	–	–	–	–	–	–	–	–	–	–	–
*Nhandu coloratovillosus* (2)	unknown origin	juv. ♂ (car. 9.1)	+	–	–	–	–	–	–	–	–	–	–	–
*Nhandu coloratovillosus* (2)	unknown origin	juv. ♂ (car. 11.2)	+	–	–	–	–	–	–	–	–	–	–	–
*Nhandu coloratovillosus* (2)	unknown origin	juv. ♂ (car. 14.3)	+	+	–	–	–	–	–	–	–	–	–	–
*Nhandu coloratovillosus* (2)	unknown origin	juv. ♂ (car. 17.0)	+	+	+	+	–	–	–	–	–	–	–	–
*Nhandu tripepii* (1)	unknown origin	♀	+	–	–	+	–	–	–	–	–	–	–	–
*Nhandu tripepii* (2)	unknown origin	juv. ♂ (car. 3.7)	+	–	–	–	–	–	–	–	–	–	–	–
*Nhandu tripepii* (2)	unknown origin	juv. ♂ (car. 6.5)	+	–	–	–	–	–	–	–	–	–	–	–
*Nhandu tripepii* (2)	unknown origin	juv. ♂ (car. 10.0)	+	–	–	–	–	–	–	–	–	–	–	–
*Nhandu tripepii* (2)	unknown origin	juv. ♂ (car. 21.0)	+	–	–	+	–	–	–	–	–	–	–	–
*Nhandu tripepii* (2)	unknown origin	♂	+	+	+	+	–	–	–	–	–	–	–	–
*Phormictopus* cf. *atrichomatus*	Dominican Republic, Barahona province	juv. (car. 3.2)	+	+	+	–	–	–	–	–	–	–	–	–
*Phormictopus* cf. *atrichomatus*	Dominican Republic, Barahona province	juv. (car. 5.6)	+	–	+	–	–	–	–	–	–	–	–	–
*Phormictopus* cf. *atrichomatus*	Dominican Republic, Barahona province	juv. (car. 8.8)	+	+	+	–	–	–	–	–	–	–	–	–
*Phormictopus auratus* (1)	Cuba, Holguín province	♀	+	–	–	+	–	–	–	–	–	–	–	–
*Phormictopus auratus* (2)	Cuba, Santiago de Cuba	♀	+	–	–	+	–	–	–	–	–	–	–	–
*Phormictopus auratus* (3)	Cuba, Holguín province	juv. ♂ (car. 16.0)	+	+	+	+	–	–	–	–	–	–	–	–
*Phormictopus auratus* (4)	Cuba, Holguín province	juv. (car. 1.9)	–	–	–	–	–	–	–	–	–	–	–	–
*Phormictopus auratus* (4)	Cuba, Holguín province	juv. (car. 3.0)	+	–	–	–	–	–	–	–	–	–	–	–
*Phormictopus cancerides*	Dominican Republic, Bahoruco province	juv. (car. 3.3)	+	–	+	–	–	–	–	–	–	–	–	–
*Phormictopus cubensis* (1)	Cuba, Pinar del Rio province	♂	+	+	+	+	–	–	–	–	–	–	–	–
*Phormictopus cubensis* (2)	Cuba, Pinar del Rio province	♀	+	–	–	+	–	–	–	–	–	–	–	–
*Phormictopus cubensis* (3)	Cuba, Pinar del Rio province	juv. (car. 1.9)	+	–	–	–	–	–	–	–	–	–	–	–
*Phormictopus cubensis* (3)	Cuba, Pinar del Rio province	juv. (car. 9.2)	+	–	+	–	–	–	–	–	–	–	–	–
*Phormictopus* sp.	Cuba, Yumurí River	♀	+	–	+	+	–	–	–	–	–	–	–	–
*Phormictopus* sp.	Cuba, Guanabo	♀	+	–	–	+	–	–	–	–	–	–	–	–
*Phrixotrichus vulpinus* (1)	Chile	♂	–	–	–	–	–	–	–	–	+	–	–	–
*Phrixotrichus vulpinus* (2)	Chile	♀	–	–	–	–	–	–	–	–	+	+	–	–
*Phrixotrichus vulpinus* (3)	Chile	juv. (car. 2.2)	–	–	–	–	–	–	–	–	+	–	–	–
*Pseudhapalopus* sp.	Colombia	juv. ♂	–	–	–	–	–	–	+	–	–	–	–	–
*Plesiopelma* sp.	Uruguay	♀	–	–	–	–	–	–	–	–	+	+	–	–
*Pterinopelma sazimai*	Brazil	juv.	+	+	–	–	–	–	–	–	–	–	–	–
*Pterinopelma sazimai*	Brazil	♂	+	+	–	+	–	–	–	–	–	–	–	–
*Reversopelma petersi*	Ecuador, Peru	♂	–	–	+	–	–	–	–	–	–	–	–	–
*Reversopelma petersi*	Ecuador, Peru	♀	+	+	–	–	–	–	–	–	–	–	–	–
*Schizopelma* sp.	Mexico, Guerrero	♂	–	–	–	–	–	–	–	–	+	–	–	–
*Sericopelma melanotarsum* (1)	Costa Rica	♀	+	+	+	+	–	–	–	–	–	–	–	–
*Sericopelma melanotarsum* (2)	Costa Rica	juv. ♂	+	+	+	–	–	–	–	–	–	–	–	–
*Sericopelma rubronitens*	Panama	♂	+	+	+	–	–	–	–	–	–	–	–	–
*Sericopelma* sp.	Costa Rica, Limon province	♀	–	+	+	–	–	–	–	–	–	–	–	–
*Sphaerobothria hoffmanni* (1)	Costa Rica, San José	♂	+	+	+	–	–	–	–	–	–	–	–	–
*Sphaerobothria hoffmanni* (2)	Costa Rica	♂	+	+	–	–	–	–	–	–	–	–	–	–
*Stichoplastoris* sp.	Costa Rica, Las Juntas	♀	+	–	–	–	–	–	–	–	–	–	–	–
*Stichoplastoris* sp.	Costa Rica, Las Juntas	♂	+	–	–	–	–	–	–	–	–	–	–	–
*Theraphosa blondi* (1)	unknown origin	♀	–	–	–	–	–	–	–	–	+	–	–	–
*Theraphosa blondi* (2)	unknown origin	juv. ♂ (car. 5.5)	–	–	–	–	–	–	–	–	+	–	–	–
*Theraphosa blondi* (2)	unknown origin	juv. ♂ (car. 6.5)	–	–	–	–	–	–	–	–	+	–	–	–
*Theraphosa blondi* (2)	unknown origin	juv. ♂ (car. 8.0)	–	–	–	–	–	–	–	–	+	–	–	–
*Theraphosa blondi* (2)	unknown origin	juv. ♂ (car. 10.0)	–	–	–	–	–	–	–	–	+	–	–	–
*Theraphosa blondi* (2)	unknown origin	juv. ♂ (car. 12.0)	–	–	–	–	–	–	–	–	+	–	–	–
*Theraphosa blondi* (2)	unknown origin	juv. ♂ (car. 14.0)	–	–	–	–	–	–	–	–	+	–	–	–
*Theraphosa blondi* (2)	unknown origin	juv. ♂ (car. 20.0)	–	–	–	–	–	–	–	–	+	–	–	–
*Theraphosa apophysis*	Venezuela, Autana River	♀	–	–	–	–	–	–	–	–	+	–	–	–
*Thrixopelma ockerti* (1)	Peru, Loreto	♀	–	–	–	–	–	–	–	–	+	+	–	–
*Thrixopelma ockerti* (2)	Peru, Loreto	juv. ♂ (car. 3.9)	–	–	–	–	–	–	–	–	–	+	–	–
*Thrixopelma ockerti* (2)	Peru, Loreto	juv. ♂ (car. 7.8)	–	–	–	–	–	–	–	–	+	+	–	–
*Thrixopelma ockerti* (2)	Peru, Loreto	juv. ♂ (car. 15.0)	–	–	–	–	–	–	–	–	+	+	–	–
*Thrixopelma ockerti* (3)	Peru, Loreto	juv. (car. 3.2)	–	–	–	–	–	–	–	–	–	+	–	–
*Vitalius paranaensis* (1)	unknown origin	♂	+	+	+	–	–	–	–	–	–	–	–	–
*Vitalius paranaensis* (2)	unknown origin	juv. (car. 5.2)	+	–	–	–	–	–	–	–	–	–	–	–

♂ = adult male; ♀ = adult female; juv. = juvenile specimen, car. = length of carapace; “+” = present; “–” = absent. The number in parenthesis following the scientific name of the particular species refers to the particular specimen in the group.

^a^ The modified type I UrS found in the holotype of *Aenigmarachne sinapophysis* Schmidt, 2005 were erroneously interpreted as type VI in the original description.

^b^ Affinity to *Citharacanthus* Pocock, 1901, the material has the same type of UrS (type I, subtype I_d_), three keels, PS, PI and A (sensu Bertani [61]), on the embolus of the male palpal bulb and separate seminal receptacles with distinct apical lobes in females but plumose stridulatory bristles on the prolateral face of trochanter I are absent. Based on personal observation, the number, size and density of stridulatory plumose bristles on trochanter I vary in different *Citharacanthus* species; e.g., *C*. *longipes* (Cambridge, 1897) has few well-developed stout bristles whereas in *C*. *meermani* Reichling & West, 2000 the bristles are distinctly smaller, narrower but denser.

^c^ Affinity to *Metriopelma* Becker, 1878, the material possesses type I urticating setae and lacks the subapical apophyses on male tibia I. The studied species share the presence of two separate seminal receptacles with extended apical lobes. The stage of this character is not known in *Metriopelma* because the female of *Metriopelma breyeri* Becker, 1878 (generic type) from Guanajuato, Mexico is still unknown. The placement of the Venezuelan and Costa Rican species in *Metriopelma* is provisional, and revision is needed.

### Ontogeny of urticating setae in Theraphosinae

We studied the ontogeny in two groups of Theraphosinae. Group A comprised the taxa *Acanthoscurria geniculata* C. L. Koch, 1841, *Brachypelma albopilosum* Valerio, 1980, *Cyrtopholis flavostriatus* Schmidt, 1995, *Eupalaestrus weijenberghi* (Thorell, 1894), *Nhandu coloratovillosus* (Schmidt, 1998), *Nhandu tripepii* (Dresco, 1984), *Phormictopus auratus* Ortiz & Bertani, 2005 and *Phormictopus cubensis* Chamberlin, 1917, with type I setae. The occurrence of UrS types in the chosen species during ontogeny is presented in Table 14. This group always showed the basic type I setae in the first nymphal stages. Subtypes I_a_ and/or I_b_ and/or I_c_ only co-occurred in the later instars, with exceptions only found in two juveniles of *Phormictopus* spp. from the Dominican Republic (car. 3.2–3.3), in which the co-occurrence of basic type I with subtypes I_a_ and I_b_ was recorded.

Group B included the taxa *Cyriocosmus perezmilesi* Kaderka, 2007, *Davus* sp. (2), *Theraphosa blondi* (Latreille, 1804), *Kochiana brunnipes* (C. L. Koch, 1842), *Phrixotrichus vulpinus* (Karsch, 1880) and *Grammostola* sp. (3) from Chile. They possessed type III or IV UrS in the first nymphal stages. The occurrence of UrS types in the selected species during their ontogeny is presented in Table 14.

In the *Cyriocosmus perezmilesi* nymph (car. 1.5; the first nymphal stage) neither UrS nor other types of abdominal setae were found in the patch where they normally are in the later instars. In the subsequent developmental stage (car. 2.2), ontogenetic precursors of the type III setae were found (Fig 11A).

In the *Davus* sp. (2) nymph (car. 2.0), an ontogenetic precursor of type III setae (0.11–0.13 long) was found, with barbs of the opposite direction and was restricted to the basal half of the shaft only. In the higher instars (car. 2.9, 4.4), a few type III setae were found that were of similar length as in the previous stage, but with the last two reversed barbs abnormally elongated and protruding as in type IV. In the next instar (car. 6.9), normal type III setae (length 0.18–0.25) were found.

In *Theraphosa blondi*, type III setae of two discrete length categories were recognised in all of the instars (car. 5.5, 6.5, 8.0, 10.0, 12.0, 14.0, 20.0 in ♂, car. 34.0 in ♀): long setae (0.28–0.32) and short setae (0.07–0.08) (Fig 12A and 12B), whose length corresponded to the length range in type IV setae. Two length categories of type III were also found in a mature female of *Kochiana brunnipes*: a visible central patch of long type III setae was bordered by a narrow band of short type III setae (Fig 2).

In the *P*. *vulpinus* juvenile (car. 2.2; UrS length 0.23–0.24) and an adult male (UrS length 0.47–0.53), only type III setae occurred in both lateral patches, type IV setae were found only in the adult female (UrS length 0.49–0.57).

In *Grammostola* sp. (3) from Chile, the early instars (car. 3.2, 3.9) possessed only type IV setae. In the later instars (car. 4.8, 5.5, 6.7), setae of intermediate morphology between types III and IV were also found. The adult male possessed both types, including intermediate forms.

The ontogeny in this group was characterised by the presence of type III or IV UrS in the early instars. In species with only type III setae (e.g., *Cyriocosmus*, *Davus*, *Hapalopus*, *Kochiana*, and *Theraphosa*), the UrS morphology was relatively constant during ontogeny. In species with types III and IV in the terminal instars, the missing type, type III or IV, as well as the UrS of intermediate morphology, appeared later during ontogeny. In the species with both types III and IV, a high degree of variability in the total length and curvature of the setae was recorded, just as in the length and diameter of the basal barbs. Both types III and IV represent the extremes of a morphological continuum.

The ontogenies of UrS types VI and VII were not studied because such material was unavailable to us.

### Systematics

Order Araneae Clerck, 1757 [62]

Infraorder Mygalomorphae Pocock, 1892 [63]

Family Theraphosidae Thorell, 1869 [2]

Subfamily Theraphosinae Thorell, 1869 [2]

Theraphosinae Thorell, 1869 –Nova Acta Regiae Societatis Scientiarum Upsaliensis, (3) 7: 161–164.

Diagnosis: The subfamily Theraphosinae distinguishes itself from all other theraphosids by the presence of abdominal urticating setae of type I or VI or VII or III and/or IV, together with a large and extended subtegulum and keels present on the embolus of the male palpal bulb (modified from Pérez-Miles et al. [18], following Perafán et al. [11]).

Distribution: North, Central and South America.

Remarks: The representatives of this subfamily can be divided into four groups according to the types of UrS possessed:

1) Group A with type I UrS present on the dorsal abdomen, including the following taxa: *Acanthoscurria*, *Aenigmarachne*, *Aphonopelma*, *Brachypelma*, *Citharacanthus*, *Clavopelma*, *Cotztetlana*, *Crassicrus*, *Cubanana*, *Cyclosternum*, *Cyrtopholis*, *Eupalaestrus*, *Lasiodora*, *Lasiodorides*, *Longilyra*, *Megaphobema*, *Metriopelma*, *Mygalarachne*, *Neischnocolus*, *Neostenotarsus*, *Nesipelma*, *Nhandu*, *Pamphobeteus*, *Phormictopus*, *Proshapalopus*, *Pseudhapalopus*, *Pterinopelma*, *Reversopelma*, *Scopelobates*, *Sericopelma*, *Sphaerobothria*, *Stichoplastoris*, *Umbyquyra*, *Vitalius*, *Xenesthis*.

2) Group B with type III and/or IV UrS present on the dorsal abdomen, including the following taxa: *Agnostopelma* (III+IV), *Aguapanela* (III+IV), *Bistriopelma* (III), *Bonnetina* (III), *Bumba* (III+IV), *Cardiopelma* (III), *Catanduba* (III), *Chromatopelma* (III+IV), *Cyriocosmus* (III), *Davus* (III), *Euathlus* (III+IV), *Grammostola* (III+IV), *Hapalopus* (III, IV), *Hapalotremus* (III), *Homoeomma* (III+IV), *Kochiana* (III; short + long version), *Magulla* (III+IV), *Magnacarina* (III), *Melloleitaoina* (III+IV), *Munduruku* (III+IV), *Phrixotrichus* (III+IV), *Plesiopelma* (III+IV), *Schizopelma* (III), *Theraphosa* (III; short + long version), *Thrixopelma* (III+IV), *Tmesiphantes* (III).

3) Group C with type VI UrS present on the dorsal abdomen, with reversions in some troglobitic species of *Hemirrhagus* [35]. The genus *Hemirrhagus* is the only representative of this group. Pérez-Miles [13] placed *Hemirrhagus* into Theraphosinae based generally on the presence of abdominal UrS, abundant leg spination and the absence of laterally extended scopulae and spatulate scopula setae, which collectively argued against its inclusion in Aviculariinae [13]. According to phylogenetic analysis proposed by Perafán et al. [11], *Hemirrhagus* is in the basal position to the rest of the tested Theraphosinae genera. The morphological similarity of type VI to type II UrS is discussed below.

4) Group D with type VII UrS present on the dorsal abdomen [11]. *Kankuamo* is the only representative of this group.

The monophyly of the groups A and B was supported by the molecular analysis carried out by Turner et al. [20], and three new tribes within Theraphosinae were proposed: Theraphosini Turner et al., 2017 (= group A with *Theraphosa*), Grammostolini Turner et al., 2017 and Hapalopini Turner et al., 2017 (= group B without *Theraphosa*). The tribe Theraphosini was represented by 18 genera (*Acanthoscurria*, *Aphonopelma*, *Brachypelma*, *Citharacanthus*, *Crassicrus*, *Cyrtopholis*, *Eupalaestrus*, *Lasiodora*, *Megaphobema*, *Nhandu*, *Pamphobeteus*, *Phormictopus*, *Sericopelma*, *Sphaerobothria*, *Stichoplastoris*, *Theraphosa*, *Vitalius*, and *Xenesthis*; 50% of known genera), and the tribes Grammostolini + Hapalopini by 12 genera (*Bonnetina*, *Bumba*, *Chromatopelma*, *Cyriocosmus*, *Davus*, *Euathlus*, *Grammostola*, *Hapalopus*, *Homoeomma*, *Phrixotrichus*, *Plesiopelma*, and *Thrixopelma*; 50% of known genera). The monophyly of the group A with type I UrS was also supported by the molecular analysis carried out by Lüddecke et al. [54]. The authors analysed nuclear and mitochondrial markers of nine genera from the group A (*Aphonopelma*, *Brachypelma*, *Crassicrus*, *Lasiodora*, *Lasiodorides*, *Megaphobema*, *Nhandu*, *Sericopelma*, and *Xenesthis*; 26% of known genera), two genera from the group B (*Grammostola* and *Kochiana*; only 8% of known genera), together with 37 taxa from other subfamilies of Theraphosidae.

Subfamily Aviculariinae Simon, 1873 [3]

Avicularinae Simon, 1873 –Mémoires de la Société Royale des Science de Liège, Bruxelles 5 (2): 1–174.

Diagnosis: The subfamily Aviculariinae distinguishes itself from all other theraphosids by the following combination of morphological characters: the presence of abdominal urticating setae of type II; legs aspinose or weakly spinose on ventral tibiae and metatarsi; scopulae on tarsi and metatarsi I and II laterally extended; the presence of separate seminal recepacles in females; the presence of one subapical apophysis or a protuberance ending in numerous spines on male tibia I, except for *Typhochlaena*, *Ybyrapora* and some *Avicularia* spp., which lack this apomorphic character [14,15] (modified from Fukushima and Bertani [14], following the concept of Aviculariinae proposed by Lüddecke et al. [54]).

Distribution: Central America and north of South America.

Genera included: *Antillena*, *Avicularia*, *Caribena*, *Iridopelma*, *Pachistopelma*, *Typhochlaena*, *Ybyrapora*.

Remarks: The monophyly of this subfamily was supported by previously published phylogenetic analyses [14,15,17,20,54,60]. This subfamily does not represent a sister group to the South American genera *Psalmopoeus*, *Tapinauchenius* and *Ephebopus* or the African genera *Stromatopelma* Karsch, 1881 and *Heteroscodra* Pocock, 1899 [15,20], as was previously proposed by West et al. [60], who transferred all of the mentioned genera to Aviculariinae, considering only well-developed and laterally extended scopulae on the tarsi and metatarsi I and II as a basic phylogenetic criterion but omitting the separation of Africa from the South American continent more than 110 Mya [64]. Later, Lüddecke et al. [54] revealed that the African genera *Stromatopelma* and *Heteroscodra* represent a sister group to African subfamily Harpactirinae Pocock, 1897 [65]. Following the concept of Aviculariinae proposed by Lüddecke et al. [54] we added the presence of type II setae to the diagnosis of Aviculariinae.

Subfamily Psalmopoeinae Samm & Schmidt, 2010 [4]

Psalmopoeinae Samm & Schmidt, 2010 –Tarantulas of the World, 142: 35–41.

Diagnosis: The subfamily Psalmopoeinae distinguishes itself from all other theraphosids by the following combination of morphological characters: the presence of two subapical apophyses on male tibia I; scopulae on tarsi and metatarsi I and II laterally extended giving a spatulate appearance; legs aspinose on tibiae and metatarsi; abdominal urticating setae are absent; the presence of separate seminal recepacles in females (modified from Hüsser [17] and Samm and Schmidt [4]).

Distribution: Central America and north of South America.

Genera included: *Psalmopoeus*, *Pseudoclamoris*, *Tapinauchenius*, *Ephebopus*.

Remarks: The monophyly of this subfamily is supported by previously published phylogenetic analyses [14,15,60], which all have a congruent topology *Ephebopus +* (*Psalmopoeus* + *Tapinauchenius*). It is also supported by molecular analyses carried out by Turner et al. [20], Lüddecke et al. [54], and Hüsser [17]. *Ephebopus* is the only genus within Psalmopoeinae, which possesses urticating setae but in contrast to abdominal urticating setae found in Theraphosinae they are located on palpal femora.

**Fig 16:**
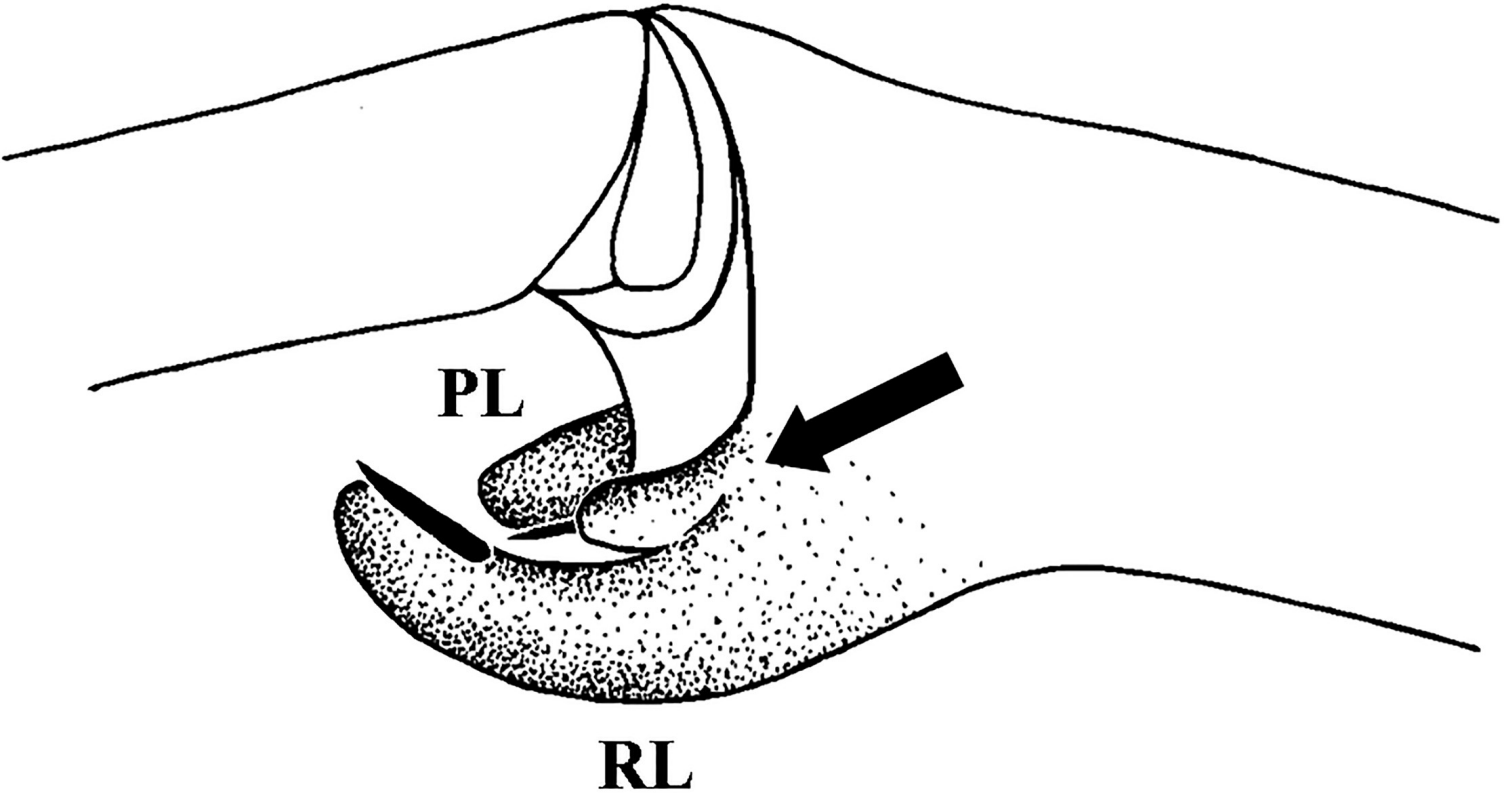
*Ephebopus rufescens* West & Marshall, 2000, male tibia I with prolateral (PL) and retrolateral (RL) tibial apophysis. The arrow shows a third additional tibial apophysis at the base of the retrolateral apophysis. Leg segments without covering setae.

Based on the study of Psalmopoeinae males herein involved we suggest a new synapomorphy consisting in having the third additional apophysis or protuberance, which is located dorsally at the base of the retrolateral tibial apophysis (Fig 16).

Subfamily Stromatopelminae Schmidt, 1993 [66], revalidated

Stromatopelminae Schmidt, 1993 –Landbuch Verlag, Hannover: 112.

Diagnosis: The subfamily Stromatopelminae distinguishes itself from all other theraphosids by the following combination of morphological characters: legs aspinose or weakly spinose; fovea circular and pit-like; the labial cuspules reduced; males without subapical apophysis on leg I (modified from Schmidt [66], Gallon [67], Gallon [68]).

Distribution: Central and Western Africa.

Genera included: *Stromatopelma*, *Heteroscodra*.

Remarks: Gallon [67] proposed the following topology for Stromatopelminae: *Encyocratella +* (*Heteroscodra* + *Stromatopelma*)). According to the phylogenetic analyses of Bertani [15] and Fukushima & Bertani [14], both based on morphological data, *Encyocratella* does not represent a sister group of *Stromatopelma + Heteroscodra*. The monophyly of clade *Stromatopelma + Heteroscodra* was confirmed by West et al. [60], Bertani [15], Fukushima & Bertani [14] and also Lüddecke et al. [54] who placed this clade as a sister group to the African subfamily Hapactirinae, respecting 110 Myr of independent evolution on both African and South American continent. Folowing the phylogenetic concept proposed by Lüddecke et al. [54], the subfamily Stromatopelminae, which was previously synonymised with Aviculariinae by West et al. [60], is hereby revalidated.

## Discussion

### Urticating setae as a defensive mechanism

The defensive strategy using urticating setae against vertebrate and invertebrate predators and intruders has been described. Two uses of UrS have been recorded: the active defence against potential intruders and the passive defence consisting in the incorporation of UrS into the silk mat for moulting or the egg sac wall, e.g., against phorid larvae (Diptera: Phoridae) [5,6,7,8,9,12]. The abdominal urticating setae of Theraphosinae emerge approximately in the first or second nymphal stage (nomenclature of instars determined after Foelix [56]). The larval stages lack abdominal UrS. The larvae do not use the defensive strategy based on the release of UrS because they live together in the egg sac and later in the security of the mother’s burrow, entirely dependent on yolk reserves. In addition, this defensive mechanism would be inefficient due to the small size and amount of UrS.

In the majority of spiders, moulting ceases at maturity. However, females of mygalomorph spiders continue to moult approximately every year throughout their lives, acquiring a fresh vestment of setae, including defensive urticating setae at each moult [6].

During the study ontogenetic development of UrS in Theraphosinae, we revealed the morphological resemblance between the airborne type III and the airborne subtypes I_b_ and I_c_ of type I setae, consisting in the presence of reversed barbs along the shaft. An explanation was proposed by Pérez-Miles [10]. Presumably, ecological pressures on the large spiders of the New World are similar, and this fact could explain the convergence of any type I subtypes with the type III morphology, probably due to their efficacy for defensive purposes [10].

### Hypotheses of evolution of urticating setae

No broadly accepted hypothesis exists despite the attention dedicated to the phylogeny of New World Theraphosidae, based on both morphological and molecular characters, from which the evolutionary hypotheses for UrS may be derived. We analysed the previously published phylogenetic hypotheses [9,11,14,15,18,20,54,60], and derived a few hypotheses of the evolution of urticating setae, complemented by a proposal of a new approach to study of UrS and their evolution.

The diagnosis of Theraphosinae proposed by Pérez-Miles et al. [18] implicitly presumes the existence of a common ancestor, from which descendants carrying abdominal UrS of types I or III and/or IV evolved.

**Fig 17:**
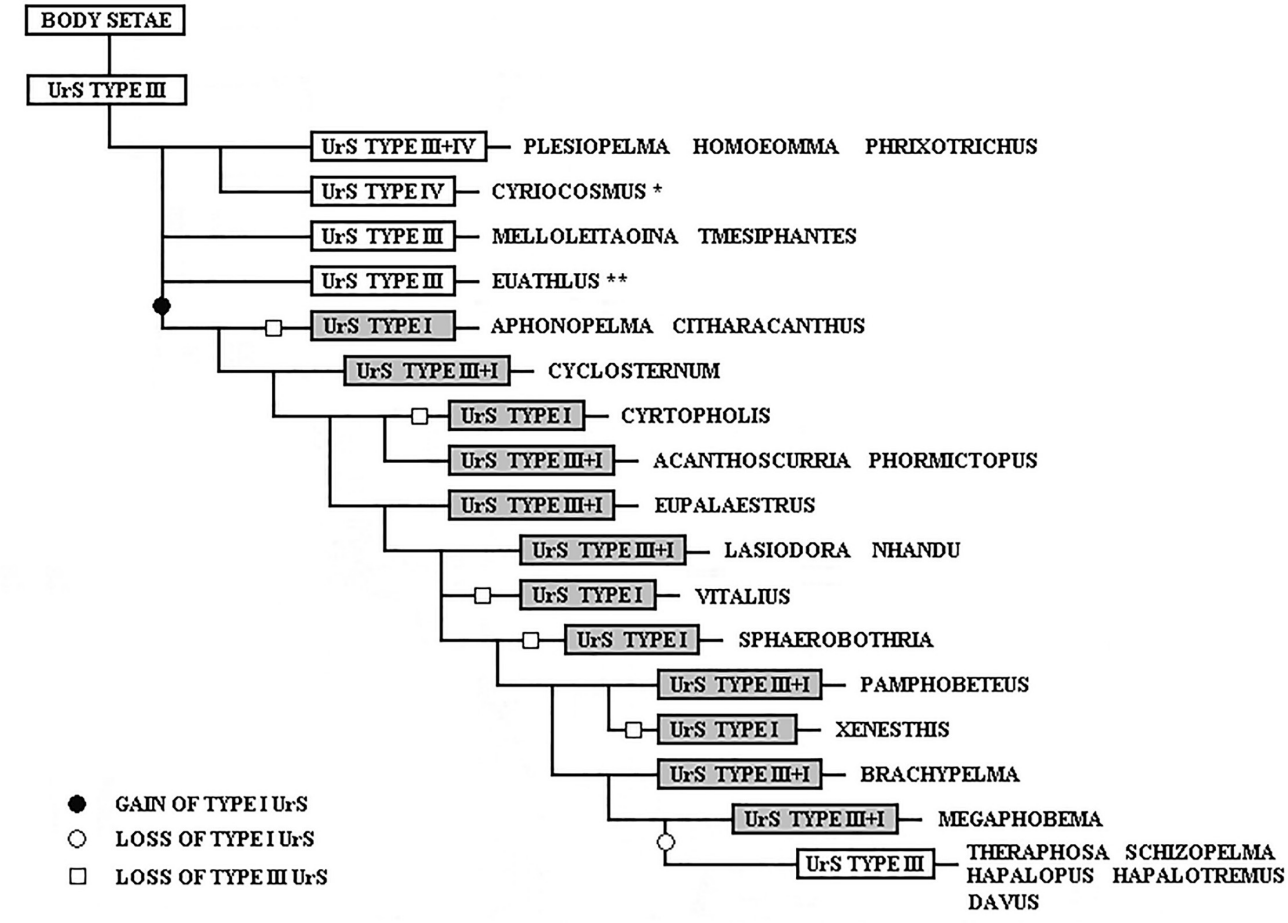
Hypothesis of urticating setae evolution based on the modified cladogram by Pérez-Miles et al. [18] and the results presented by Bertani & Guadanucci [9]. Remarks: * *Cyriocosmus* possesses only type III setae instead of type IV. ** In *Euathlus*, type III setae occur together with type IV.

A hypothesis of UrS evolution derived from the Theraphosinae phylogeny published by Pérez-Miles et al. [18] and Bertani & Guadanucci [9] is proposed (Fig 17). The genera with type I setae compose a monophyletic group, together with the ingroup *Theraphosa* + (*Schizopelma* + (*Davus* sensu Gabriel [19] + (*Hapalopus* + *Hapalotremus*))) with only type III UrS and unipartite spermatheca; a loss of type I UrS during evolution is presumed.

**Fig 18:**
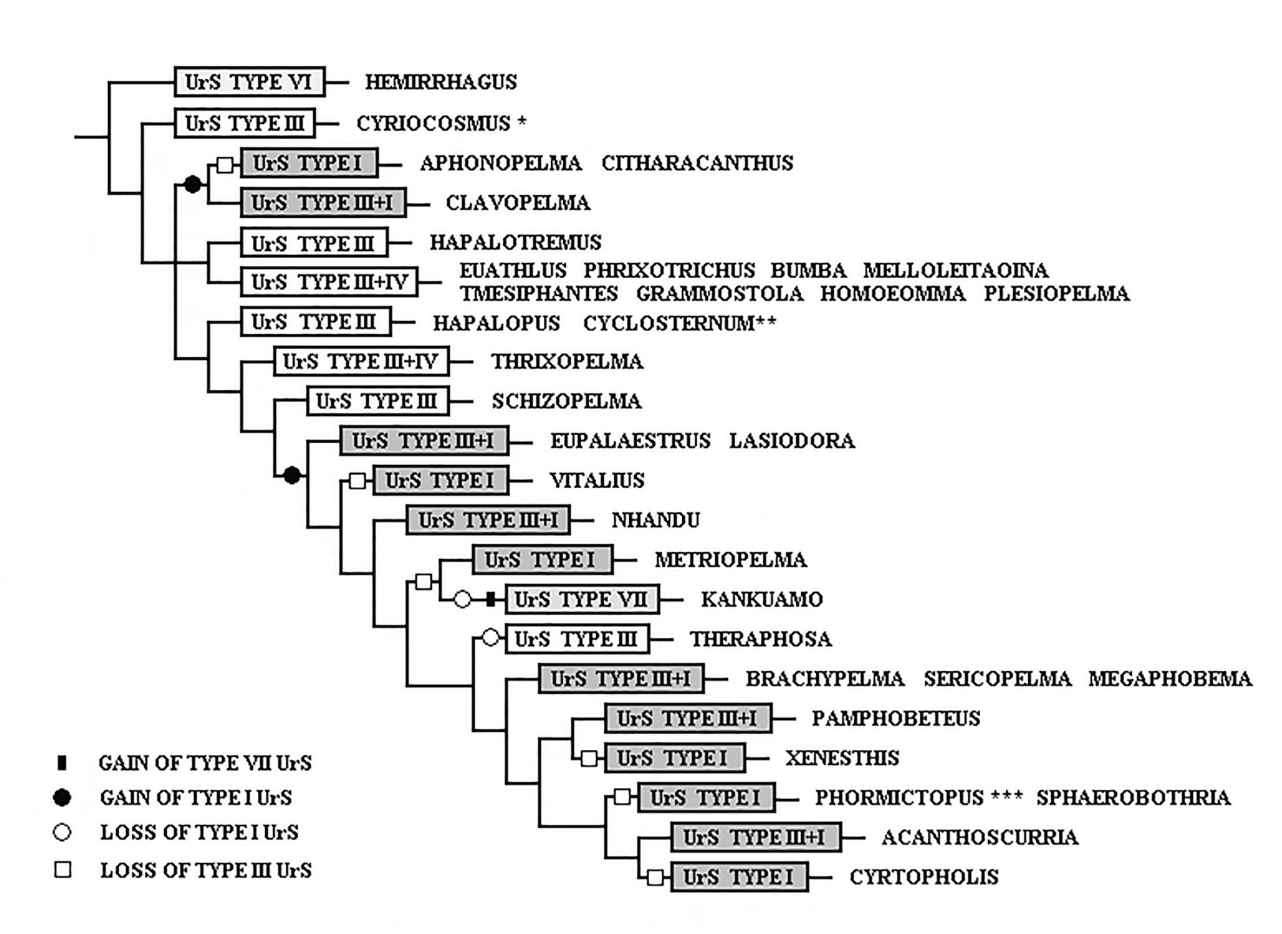
Hypothesis of urticating setae evolution based on the modified cladogram by Perafán et al. [11]. Remarks: * In the data matrix, *Cyriocosmus* is mentioned without any type of urticating setae instead of the presence of type III setae. This could explain its basal position in the cladogram. ** Female syntype of *Cyclosternum schmardae* as generic type possesses type I setae. *** *Phormictopus* possesses urticating setae of types I and III.

Another hypothesis of UrS evolution (Fig 18), which is derived from the phylogenetic analysis performed by Perafán et al. [11], indicates, among others, that the type VII UrS of *Kankuamo* evolved from type I, that *Theraphosa* lost its type I UrS during evolution and that *Hemirrhagus* represents a basal group to the rest of the tested Theraphosinae genera. The taxa with type I UrS comprise two non-sister groups, in which the independent evolution of type I setae is supposed.

Bertani & Guadanucci [9] suggested that the UrS of types II, V and III had evolved independently, as it was later confirmed by Lüddecke et al. [54], and that the types I and IV had evolved later from an ancestor type III setae; type VI was neither studied nor discussed. This hypothesis was supported by the differences in position, structure and the release mechanism of UrS and by the existence of intermediates between types I and III, and III and IV. However, no intermediates between types I and IV were found [9]. Bertani & Guadanucci [9] hypothesised that urticating setae of type II and type III had independently evolved from the relevant body setae and noted 1) the identical manner in which they are inserted into the spider tegument (Bertani & Guadanucci [9]: Figs 23 and 24), 2) the resemblance between the truncated basal part of body setae and stalks in UrS, and 3) the morphological similarity of basal barbs in some variants of body setae and UrS. They found body setae variants with either type II or III urticating setae, but not with types I and type IV [9]. Unfortunately, this theory does not offer an explanation of 1) how the morphologically more complicated type I setae evolved from the ancestor type III setae, whose morphology is distinctly different (the differences are mainly due to the presence of basal barbs and their arrangement into three longitudinal rows), 2) why the ancestor type III setae evolved into two different types of urticating setae, 3) why the intermediates between type I and type IV does not exist, if both types evolved from the common ancestor type III setae and 4) why the authors rejected the hypothesis proposed Pérez-Miles [10] that type III setae in taxa with and without type I setae represent two different kinds of non-homologous setae masked by surface similarity. In Fig 15, Bertani & Guadanucci [9] marked by arrow the type I seta intermixed with type III setae. However, their “type III setae” also have basal “reversed” barbs (“reversed” sensu Cooke et al., 1972, “basal” sensu revision in the present study). For that reason they are misinterpreted as type III setae. These basal “reversed” barbs in type III setae have never been recorded in taxa with type III and III+IV. If type III setae evolved from body setae, another conflict remains. Type III setae in taxa with type I were derived from type I morphology during ontogeny and so they can not be derived from two different morphological structures. If yes, it is an example of homoplasy. In agreement with Bertani & Guadanucci [9] we did not find body setae variants with type I so we could not compare a morphology of body setae in taxa with and without type I setae.

**Fig 19:**
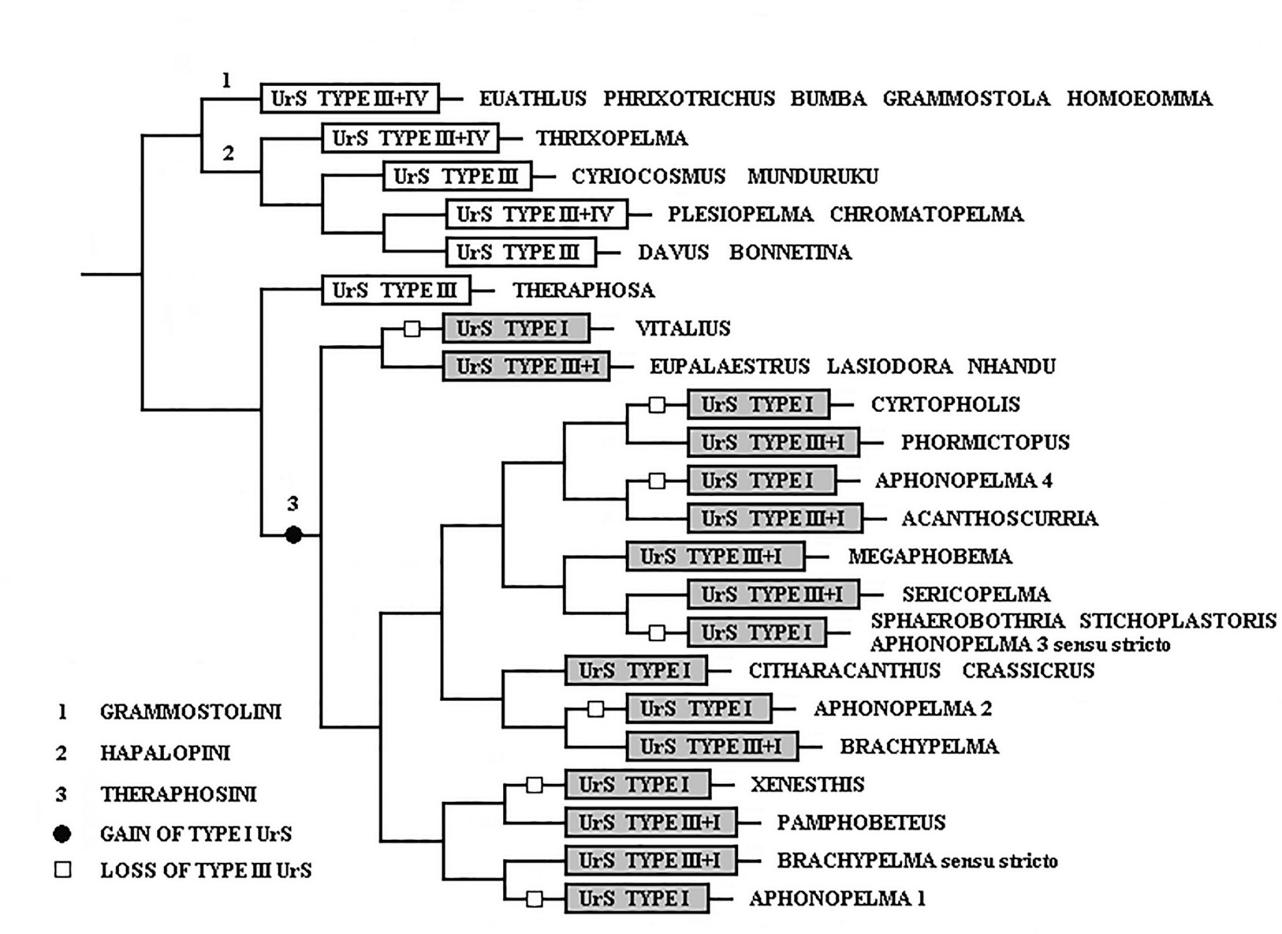
Hypothesis of urticating setae evolution based on the modified cladogram by Turner et al. [20]. Taxa with type I setae represent a monophyletic group.

In 2017, Turner et al. [20] used a fragment of mitochondrial genome for the first time to build a gene tree for the inference of a relationships within the family of Theraphosidae (Turner et al. [20]: Figs 2–5), with the following impacts to a contemporary systematics. They recognised three monophyletic groups within Theraphosinae: the tribe Theraphosini Turner et al., 2017, comprising taxa with type I UrS including *Theraphosa* with only type III UrS, and another two tribes Hapalopini Turner et al., 2017 and Grammostolini Turner et al., 2017, with UrS of type III or III+IV. The basal position of *Theraphosa* within Theraphosini may be clarified by the addition of further molecular data, both from unsampled genera and new fragments of mitochondrial or nuclear genome. In comparison to the previously published evolutionary hypotheses of UrS phylogeny (Figs 17 and 18), the hypothesis proposed by Turner et al. [20] (Fig 19) is the most parsimonious, minimising the total number of character-state changes because the taxa with type I UrS comprise a monophyletic group and whatever loss of type I urticating setae during evolution (Fig 16) or a gain of type I setae by two paraphyletic groups (Fig 17) are not supposed.

Bertani & Guadanucci [9] recognised three different types of body setae: a) long tactile setae, b) intermediate-length highly plumose setae and c) short body setae, from which the UrS probably evolved. Studying the abdominal setae in *Grammostola* sp. from Brazil (Bertani & Guadanucci [9]: Fig 25), the authors presumed that type III UrS had evolved from finely plumose body setae. In the same specimen, they also found another type of short body seta (Bertani & Guadanucci [9]: Fig 18), that was non-plumose and 0.16 long, with long barbs along one side of the shaft. The length of these setae and the uniform orientation of barbs coincided with the length range of type IV UrS and their arrangement of barbs.

In comparison to both types of short body setae as precursors of UrS found in *Grammostola* sp. [9], we found body setae of different morphology in two Aviculariinae, *Antillena rickwesti* (Fig 10) and *Iridopelma hirsutum* (Fig 9B). We suppose that the fusion of barbs with the shaft may lead to the morphology of type II setae. As the type II setae of Aviculariinae evolved independently to the UrS of Theraphosinae and both subfamilies represent two non-sister groups [54], this should explain the differences in the morphology of body setae in Aviculariinae and Theraphosinae.

Concerning the subfamily Theraphosinae, the only way to reconstruct its phylogeny and to infer the evolution of UrS, is to perform a thorough phylogenomic analysis comprising taxa with all known types of UrS (I, III, III+IV, VI and VII). At the same time, we also propose a new approach to the study of abdominal UrS evolution in Theraphosinae. If type II setae and type III setae were derived from morphologically different body setae, this would indicate that all types of abdominal UrS, probably including types VI and VII, evolved from any type of body setae. If UrS evolved from the same type of body setae but in different ways, then they should be considered homologous. To better understand the evolutionary process consisting of morphological specialisations of body setae and resulting in different types of defensive setae (UrS) in Theraphosinae, it seems crucial to recognise, from which morphological types of body setae different types of UrS evolved and to understand how it occurred. The existence of such types of body setae is presumed based on the existence of already documented intermediates between the particular type of UrS (II or III) and the corresponding body setae.

## Conclusions

The morphology of UrS was studied on 144 taxa of New World theraphosids, including ontogenetic stages in chosen species, except for species with type VII UrS. Four different types of ontogenetic development of abdominal UrS were recognised within Theraphosinae, two of which were studied in detail. The typology of UrS was revised, and types I, III and IV were redescribed. The UrS in spiders with type I setae, which were originally among type III or were considered setae of intermediate morphology between types I and III, are now considered to be ontogenetic derivatives of type I and its modified forms are described as subtypes. The new terminology coincides with the hypothesis proposed by Pérez-Miles (2002) and also with the phylogenetic hypotheses proposed by Turner et al. [20] (Fig 19), and Lüddecke et al. [54], presenting the genera with type I UrS as a monophyletic group within Theraphosinae for the first time, and the presence of type I setae as a unique synapomorphy of this group. Setae of intermediate morphology between that of body setae and type II urticating setae that were found in *Iridopelma hirsutum* and *Antillena rickwesti* may provide another evidence that type II urticating setae evolved from body setae. We suppose that the fusion of barbs with the shaft may lead to the morphology of type II setae. As the type II setae of Aviculariinae evolved independently to the UrS of Theraphosinae and both subfamilies represent two non-sister groups [54], this should explain the differences in the morphology of body setae in Aviculariinae and Theraphosinae. The terminology of “barbs” and “reversed barbs” was revised and redefined, newly emphasizing the real direction of barbs.

## Supporting information

S1 File List of examined material, voucher numbers and location information. (DOC)
