# Supplementary material for: Urticating setae of tarantulas (Araneae: Theraphosidae): Morphology, revision of typology and terminology and implications for taxonomy
Source: PLoS One. 2019 Nov 11;14(11):e0224384. doi: 10.1371/journal.pone.0224384 (PMC6844489; doi:10.1371/journal.pone.0224384)
Supplement: S1 File — (DOC) [file pone.0224384.s001.doc]

**Supporting information**

**S1 File. Material examined.**

ISCHNOCOLINAE

*Holothele* sp., ♂ (NMPC P6d-57/2018), Peru, Rio Napo; *Holothele* sp., ♂ (NMPC P6d-57/2018), Venezuela, State of Aragua; *Holothele* sp., ♂ (NMPC P6d-57/2018), ♀ (NMPC P6d-57/2018), Venezuela, State of Bolívar, San Isidro; *Holothele* sp., ♂ (car. 11.3) (NMPC P6d-57/2018), Venezuela, State of Carabobo;

Voucher material of Ischnocolinae deposited in the NMPC under inventory number P6d-57/2018.

SCHISMATOTHELINAE

*Euthycaelus colonicus* Simon, 1889, ♂ (NMPC P6d-58/2018), Venezuela, State of Carabobo, Puerto Cabello;

*Neoholothele* sp., ♀ (NMPC P6d-58/2018), Colombia; *Neoholothele* sp., ♀ (car. 8.0) (NMPC P6d-58/2018), ♂ (NMPC P6d-58/2018), Venezuela, Isla Margarita;

*Schismatothele* sp., ♂ (NMPC P6d-58/2018), Venezuela, State of Mérida (SW); *Schismatothele* sp., ♂ (NMPC P6d-58/2018), Venezuela, State of Bolívar; *Schismatothele* sp., ♂ (NMPC P6d-58/2018), ♀ (NMPC P6d-58/2018), Venezuela, State of Guarico, Altagracia de Orituco; *Schismatothele* sp., ♂ (NMPC P6d-58/2018), Venezuela, State of Aragua, Maracay;

Voucher material of Schismatothelinae is deposited in the NMPC under inventory number P6d-58/2018.

PSALMOPOEINAE

*Ephebopus cyanognathus* West & Marshall, 2000, juv. ♀ (car. 6.0) (NMPC P6d-59/2018), French Guyana; *Ephebopus rufescens* West & Marshall, 2000, ♀ (NMPC P6d-59/2018), ♂ (NMPC P6d-59/2018), French Guyana;

*Psalmopoeus cambridgei* Pocock, 1895, ♂ (NMPC P6d-59/2018), without further data; *Psalmopoeus irminia* Saager, 1994, ♀ (NMPC P6d-59/2018), unknown origin; *Psalmopoeus reduncus* (Karsch, 1880), ♂ (NMPC P6d-59/2018), unknown origin;

*Tapinauchenius* sp., ♀ (NMPC P6d-59/2018), juv. (car. 2.2) (NMPC P6d-59/2018), Colombia, Puerto Arica; *Tapinauchenius* sp., ♀ (NMPC P6d-59/2018), Venezuela, State of Delta Amacuro; *Tapinauchenius* sp., ♂ (NMPC P6d-59/2018), Peru;

Voucher material of Psalmopoeinae is deposited in the NMPC under inventory number P6d-59/2018.

AVICULARIINAE

*Antillena rickwesti* Bertani & Huff, 2013, ♀ (NMPC P6d-60/2018), ♂ (NMPC P6A 6062), Dominican Republic, Pedernales province; *Avicularia hirschii* Bullmer, Thierer-Lutz & Schmidt, 2006, ♀ (NMPC P6d-60/2018), juv. (car. 3.0) (NMPC P6d-60/2018), Ecuador; *Avicularia* sp., ♀ (NMPC P6d-60/2018), Bolivia, Reyes, Beni province; *Avicularia* sp., ♂ (NMPC P6d-60/2018), Venezuela; *Avicularia* sp., ♀ (NMPC P6d-60/2018), Peru, Puerto Maldonado, Rio Madre de Dios; *Caribena versicolor* (Walckenaer, 1837), ♂ (1) (NMPC P6d-60/2018), juv. (2) (car. 2.5) (NMPC P6d-60/2018), juv. (3) (car. 3.5) (NMPC P6d-60/2018), unknown origin;

*Iridopelma hirsutum* Pocock, 1901, juv. ♀ (car. 6.5) (NMPC P6d-60/2018), Brazil, Pernambuco, Recife;

*Pachistopelma bromelicola* Bertani, 2012, ♀ (NMPC P6d-60/2018), Brazil;

Voucher material of Aviculariinae is deposited in the NMPC under inventory number P6d-60/2018.

THERAPHOSINAE

*Acanthoscurria geniculata* (C. L. Koch, 1842), ♀ (NMPC P6d-61/2018), juv. (car. 1.7, 6.0, 7.3) (NMPC P6d-61/2018), without further data; *Acanthoscurria* sp., ♀ (NHMW 135), Paraguay, Terr. Fonciere; *Acanthoscurria* sp., ♀ (NMPC P6d-61/2018), juv. (car. 1.7) (NMPC P6d-61/2018), Peru, Madre de Dios province, Puerto Maldonado; *Acanthoscurria suina* Pocock, 1903, ♂ (NMPC P6d-61/2018), Uruguay; *Acanthoscurria theraphosoides* (Doleschall in Ausserer, 1871), ♂ holotype (NHMW 146), Brazil; *Aenigmarachne sinapophysis* Schmidt, 2005, ♂ holotype (SMFD 57342), Costa Rica, Guanacaste province; *Agnostopelma gardel* Pérez-Miles & Weinmann, 2010, juv. (car. 2.8) (NMPC P6d-61/2018), Colombia, Boyacá Department, Belen; *Aphonopelma bicoloratum* Struchen, Brändle & Schmidt, 1996, juv. ♂ (1) (car. 7.0, 10.0, 14.5) (NMPC P6d-61/2018), ♂ (2) (car. 15.0) (NMPC P6d-61/2018), Mexico; *Aphonopelma crinirufum* (Valerio, 1980), ♀ (NMPC P6d-61/2018), Costa Rica, Puntarenas, ♂ (NMPC P6d-61/2018), Costa Rica, Peninsula de Nicoya; *Aphonopelma seemanni* (Cambridge, 1897), ♀ (NMPC P6d-61/2018), Costa Rica, ♂ (NMPC P6d-61/2018), Costa Rica, Alajuela province;

*Bistriopelma lamasi* Kaderka, 2015, ♂ holotype (MUSM-ENT 0506555), ♀ paratype (MUSM-ENT 0507717), Peru, Ayacucho, Pampa Galeras, 4.000 m a.s.l.; *Bistriopelma matuskai* Kaderka, 2015, ♀ holotype (MUSM-ENT 0507716), ♂ paratype (MUSM-ENT 0511171), Peru, Apurímac province, Abancay, Nevado de Ampay, 4.003 m a.s.l.; *Bistriopelma* sp., ♀ (MUSM-ENT 0511177), Peru, Puno, Isla Amantani; *Bonnetina rudloffi* Vol, 2001, ♀ (NMPC P6d-61/2018), Mexico; *Bonnetina* sp. (probably *B. tanzeri* Schmidt, 2012), ♂ (NMPC P6d-61/2018), Mexico; *Brachypelma albiceps* Pocock, 1903, ♀ (NMPC P6d-61/2018), unknown origin; *Brachypelma albopilosum* Valerio, 1980, juv. (1) (car. 2.1) (NMPC P6d-61/2018), juv. ♀ (2) (car. 14.0) (NMPC P6d-61/2018), unknown origin; *Brachypelma auratum* Schmidt, 1992, ♀ (NMPC P6d-61/2018), Mexico; *Brachypelma baumgarteni* Smith, 1993, ♀ (NMPC P6d-61/2018), ♂ (NMPC P6d-61/2018), Mexico; *Brachypelma fossorium* Valerio, 1980, ♂ (NMPC P6d-61/2018), Costa Rica, Guanacaste province, coastal line; *Brachypelma klaasi* (Schmidt & Krause, 1994), ♀ (NMPC P6d-61/2018), ♂ (NMPC P6d-61/2018), Mexico; *Brachypelma smithi* (Cambridge, 1897), juv. ♀ (car. 21.0) (NMPC P6d-61/2018), unknown origin; *Brachypelma* sp., ♂ (NMPC P6d-61/2018), Mexico, Oaxaca; *Brachypelma verdezi* Schmidt, 2003, ♀ (NMPC P6d-61/2018), ♂ (NMPC P6d-61/2018), Mexico;

*Chromatopelma cyanopubescens* Schmidt, 1995, ♀ (1) (NMPC P6d-61/2018), ♂ (2) (NMPC P6d-61/2018), juv. (3) (car. 3.0, 4.1, 5.0, 5.9, 7.7, 9.5, 10.6, 12.4, 14.0, 17.3, 18.8.) (NMPC P6d-61/2018), juv. (4) (car. 3.0, 4.0, 5.0, 6.0, 7.5, 9.2, 10.5, 13.2, 15.0, 16.5 ♂) (NMPC P6d-61/2018), juv. (5) (car. 4.5) (NMPC P6d-61/2018), Venezuela; *Citharacanthus cyaneus* (Rudloff, 1994), ♂ (NMPC P6d-61/2018), Cuba, Granma; *Citharacanthus livingstoni* Schmidt & Weinmann, 1996, ♂ holotype (SMFD 38559), Guatemala, Livingston; *Citharacanthus longipes* (Cambridge, 1897), ♀ (NMPC P6d-61/2018), ♂ (NMPC P6d-61/2018), Mexico, State of Chiapas; *Citharacanthus* sp., ♂ (NMPC P6d-61/2018), Mexico, State of Veracruz, Catemaco; affinity to *Citharacanthus*, ♂ (1) (NMPC P6d-61/2018), juv. ♀ (2) (car. 2.1, 10.5, ♀) (NMPC P6d-61/2018), Costa Rica, Limón, Guapiles; *Crassicrus lamanai* Reichling & West, 1996, ♀ (NMPC P6d-61/2018), unknown origin; *Cyclosternum schmardae* Ausserer, 1871, ♀ syntype (NHMW 110), Ecuador, Cordillera; *Cyclosternum* sp., ♀ (MUSM-ENT 0507718), Peru, Madre de Dios province, Puerto Maldonado; *Cyclosternum* sp., ♀ (MUSM-ENT 0503327), Peru, Ucayali, Pucallpa; *Cyriocosmus leetzi* Vol, 1999, ♂ (NMPC P6d-61/2018), Venezuela, State of Táchira, San Cristobal; *Cyriocosmus perezmilesi* Kaderka, 2007, ♂ (1) (NMPC P6d-61/2018), ♀ (2) (NMPC P6d-61/2018), juv. (3) (car. 1.5, 2.2) (NMPC P6d-61/2018), Bolivia, Beni province; *Cyriocosmus rogerioi* Pérez-Miles & Weinmann, 2009, ♂ holotype (SMFD 60239), Peru, Kuelap near Chachapoyas; *Cyriocosmus venezuelensis* Kaderka, 2010, ♂ holotype (NMPC P6E-2997), ♀ paratype (NMPC P6E-2999), Venezuela, State of Lara, Carora; *Cyrtopholis flavostriatus* Schmidt, 1995, ♀ holotype (1) (SMFD 38507, incorrectly labeled as ♂), Lesser Antilles, Guano, juv. ♀ (2) (car. 7.5, 18.0) (NMPC P6d-61/2018), unknown origin; *Cyrtopholis* sp., ♂ (NMPC P6d-61/2018), Cuba, Guantánamo; *Cyrtopholis* sp., ♂ (NMPC P6d-61/2018), Cuba, Holguín province, coastal line; *Cyrtopholis* sp., ♂ (1) (NMPC P6d-61/2018), subadult ♂ (NMPC P6d-61/2018), Cuba, Santiago de Cuba province, Baconao, coastal line; *Cyrtopholis* sp., ♂ (2) (NMPC P6d-61/2018), Cuba, Santiago de Cuba province, Baconao, coastal line; *Cyrtopholis* sp., ♀ (NMPC P6d-61/2018), Cuba, Sanctí Spíritus province, Trinidad; *Cyrtopholis* sp., ♀ (NMPC P6d-61/2018), Dominican Republic, La Vega province, Cordillera Central; *Cyrtopholis* sp., ♀ (1) (NMPC P6d-61/2018), juv. (2) (car. 2.7) (NMPC P6d-61/2018), Dominican Republic, Pedernales province, Sabana de Sansón;

*Davus pentaloris* (Simon, 1888), ♀ (NMPC P6d-61/2018), Mexico, State of Oaxaca, costal line; *Davus ruficeps* (Simon, 1891), ♂ (NMPC P6d-61/2018), Costa Rica, Peninsula de Nicoya; *Davus* sp., ♂ (1) (NMPC P6d-61/2018), juv. ♂ (2) (car. 2.0, 2.9., 4.4, 5.6, 6.9, 7.8, 9.0, 11.0, 13.0, 14.6 ♂) (NMPC P6d-61/2018), unknown origin;

*Euathlus* sp., ♂ (NMPC P6d-61/2018), Chile; *Euathlus* sp., ♀ (NMPC P6d-61/2018), Chile, Volcán Chillán; *Euathlus truculentus* Ausserer, 1875, ♂ (NMPC P6d-61/2018), Chile, Santiago de Chile - Valparaiso; *Eupalaestrus larae* Ferretti & Barneche, 2012, ♀ (NMPC P6d-61/2018), Argentina; *Eupalaestrus weijenberghi* (Thorell, 1894), ♂ (1) (NMPC P6d-61/2018), ♀ (2) (NMPC P6d-61/2018), juv. ♀ (3) (car. 4.2, 6.8, 10.2, 11.7) (NMPC P6d-61/2018), Uruguay;

*Grammostola pulchripes* Simon, 1891, juv. ♀ (car. 12.0) (NMPC P6d-61/2018), unknown origin; *Grammostola (Lasiopelma) grossa* (Ausserer, 1871), ♂ holotype (NHMW 138), Brazil; *Grammostola* sp., ♂ (1) (NMPC P6d-61/2018), juv. (2) (car. 3.0) (NMPC P6d-61/2018), juv. ♂ (3) (car. 3.2, 3.9, 4.8, 5.5, 6.7) (NMPC P6d-61/2018), Chile; *Grammostola* sp., ♂ (4) (NMPC P6d-61/2018), Chile; *Grammostola* sp., ♀ (NMPC P6d-61/2018), Argentina;

*Hapalopus butantan* (Pérez-Miles, 1998), ♀ (INPA 0615), Brazil, Amazonas, Presidente Figueiredo, Hidrelétrica de Balbina (Ilha Massaranduba); *Hapalopus formosus* Ausserer, 1875, ♂ holotype (NHMW 112), Colombia, Bogotá; *Hapalopus triseriatus* Caporiacco, 1955, ♀ (NMPC P6d-61/2018), Venezuela, State of Mérida; *Hapalopus* sp., ♂ (NMPC P6d-61/2018), juv. (car. 1.8) (NMPC P6d-61/2018), Venezuela, State of Lara; *Hapalopus* sp., ♀ (NMPC P6d-61/2018), ♂ (NMPC P6d-61/2018), Costa Rica; *Hapalotremus* sp., ♀ (NMPC P6d-61/2018), Peru; *Hapalotremus* sp., ♀ (MUSM-ENT 0511168), Peru, Cusco, Tipon; *Hapalotremus* sp., ♀ (MUSM-ENT 0511150), Peru, Cusco, Ollantaytambo; *Hapalotremus* sp., ♀ (car. 1.61, 1.64, 14.1) (MUSM-ENT 0513032), Peru, Cusco, Calca, Pitusiray; *Hemirrhagus coztic* Pérez-Miles & Locht, 2003, ♀ (UNAM), Mexico, Morelos, Tepoztlán, Cueva del Diablo; *Hemirrhagus eros* Pérez-Miles & Locht, 2003, ♀ (UNAM), Mexico, Oaxaca, El Punto; *Hemirrhagus ocellatus* Pérez-Miles & Locht, 2003, exuvia (UNAM), Mexico, Estado de Mexico, Cueva Peña Blanca; *Hemirrhagus papalotl* Pérez-Miles & Locht, 2003, ♀ (UNAM), Mexico, Guerrero, Gruta de Aguacachil, Taxco, juv. ♂ (car. 9.0) (NMPC P6d-61/2018), Mexico, Guerrero, Cave La Joya; *Homoeomma* sp., ♂ (NMPC P6d-61/2018), ♀ (NMPC P6d-61/2018), Chile;

*Kochiana brunnipes* (C. L. Koch, 1842), ♀ (1) (NMPC P6d-61/2018), juv. ♀ (2) (car. 1.0, 3.1, 10.3) (NMPC P6d-61/2018), unknown origin;

*Lasiodora (Crypsidromus) isabellina* (Ausserer, 1871), juv. ♀ holotype (NHMW 109), Brazil, Rio de Janeiro;

*Magnacarina* sp., ♂ holotype (UNAM), Mexico, Oaxaca, Bahías de Huatulco, Crucesita; *Megaphobema mesomelas* (Cambridge, 1897), ♂ (NMPC P6d-61/2018), unknown origin; *Megaphobema robustum* (Ausserer, 1875), ♀ (NMPC P6d-61/2018), unknown origin; *Megaphobema velvetosoma* Schmidt, 1995, ♀ (NMPC P6d-61/2018), Ecuador; *Metriopelma* sp., ♂ (NMPC P6d-61/2018), ♀ (NMPC P6d-61/2018), Costa Rica, Alajuela province, Brasilia; *Metriopelma* sp., ♀ (NMPC P6d-61/2018), ♂ (NMPC P6d-61/2018), Venezuela, Isla Margarita; *Metriopelma* sp., juv. (car. 7.5) (NMPC P6d-61/2018), Venezuela, State of Barinas, Ciudad Bolivia near Barinas; *Metriopelma* sp., juv. (car. 9.0) (NMPC P6d-61/2018), Venezuela, State of Aragua, Ocumare de la Costa near Maracay; *Metriopelma* sp., ♂ (NMPC P6d-61/2018), Venezuela, State of Portuguese; *Mygalarachne brevipes* Ausserer, 1871, ♀ holotype (NHMW 139), Honduras;

*Neischnocolus armihuariensis* (Kaderka, 2014), ♂ holotype (MUSM-ENT 0506547), Peru, Cuzco province, Armihuari; *Neischnocolus* sp., ♂ (NMPC P6d-61/2018), Colombia, Ibaque Tolima; *Neischnocolus* sp., ♂ (NMPC P6d-61/2018), Costa Rica; *Neischnocolus* sp., ♀ (NMPC P6d-61/2018), Ecuador, frontier between provinces of Imbabura and Carchi; *Neischnocolus* sp., ♀ (NMPC P6d-61/2018), Venezuela, State of Guarico, Altagracia de Orituco; *Neischnocolus weinmanni* Pérez-Miles, 2008, ♂ holotype, Venezuela, Apure, La Azulita (Pérez-Miles et al., 2008); *Nhandu coloratovillosus* (Schmidt, 1998), ♀ (1) (NMPC P6d-61/2018), juv. (2) (car. 3.7, 6.0, 7.4, 9.1, 11.2, 14.3., 17.0) (NMPC P6d-61/2018), unknown origin; *Nhandu tripepii* (Dresco, 1984), ♀ (1) (NMPC P6d-61/2018), juv. ♂ (2) (car. 3.7, 6.5, 10.0, 21.0, ♂) (NMPC P6d-61/2018), unknown origin;

*Phormictopus* cf. *atrichomatus* Schmidt, 1991, juv. (car. 3.2, 5.6, 8.8) (NMPC P6d-61/2018), Dominican Republic, Barahona province, Sierra de Bahoruco, Cabral; *Phormictopus auratus* Ortiz & Bertani, 2005, ♀ (1) (NMPC P6d-61/2018), juv. ♂ (3) (car. 16.0) (NMPC P6d-61/2018), juv. (4) (car. 1.9, 3.0) (NMPC P6d-61/2018), Cuba, Holguín province, ♀ (2) (car. 26.3) (NMPC P6d-61/2018), Cuba, Santiago de Cuba; *Phormictopus* *cancerides* (Latreille, 1806), juv. (car. 3.3) (NMPC P6d-61/2018), Dominican Republic, Bahoruco province, Sierra de Neiba, Neiba; *Phormictopus* *cubensis* Chamberlin, 1917, ♂ (1) (NMPC P6d-61/2018), ♀ (2) (NMPC P6d-61/2018), juv. (3) (car. 1.9, 9.2) (NMPC P6d-61/2018), Cuba, Pinar del Rio province, Soroa; *Phormictopus* sp., ♀ (NMPC P6d-61/2018), Cuba, Guantánamo province, Yumurí River; *Phormictopus* sp., ♀ (NMPC P6d-61/2018), Cuba, Ciudad de La Habana province, Guanabo; *Phrixotrichus vulpinus* (Karsch, 1880), ♂ (1) (NMPC P6d-61/2018), ♀ (2) (NMPC P6d-61/2018), juv. (3) (car. 2.2) (NMPC P6d-61/2018), Chile; *Plesiopelma* sp., ♀ (NMPC P6d-61/2018), Uruguay; *Pseudhapalopus* sp., juv. ♂ (car. 10.5) (NMPC P6d-61/2018), Colombia; *Pterinopelma sazimai* Bertani, Nagahama & Fukushima, 2011, juv. (car. 2.1) (NMPC P6d-61/2018), ♂ (NMPC P6d-61/2018), Brazil;

*Reversopelma petersi* Schmidt, 2001, ♂ holotype (SMFD 39991), ♀ paratype (SMFD 56737), Ecuador, Peru;

*Schizopelma* sp., ♂ (NMPC P6d-61/2018), Mexico, Guerrero, coastal line; *Sericopelma melanotarsum* Valerio, 1980, ♀ (1) (NMPC P6d-61/2018), Costa Rica, subadult ♂ (2) (NMPC P6d-61/2018), Costa Rica; *Sericopelma rubronitens* Ausserer, 1875, ♂ holotype (NHMW 154), Panama; *Sericopelma* sp., ♀ (NMPC P6d-61/2018), Costa Rica, Limon province; *Sphaerobothria hoffmanni* Karsch, 1879, ♂ (1) (NHMW 142), Costa Rica, San José; ♂ (2) (NMPC P6d-61/2018), Costa Rica; *Stichoplastoris* sp., ♀ (NMPC P6d-61/2018), ♂ (NMPC P6d-61/2018), Costa Rica, Guanacaste province, Las Juntas;

*Theraphosa blondi* Thorell, 1870, ♀ (1) (car. 34.0) (NMPC P6d-61/2018), juv. ♂ (2) (car. 5.5, 6.5, 8.0, 10.0, 12.0, 14.0, 20.0) (NMPC P6d-61/2018), unknown origin; *Theraphosa apophysis* (Tinter, 1991), ♀ (car. 28.8) (NMPC P6d-61/2018), Venezuela, State of Amazonas, Autana River; *Thrixopelma ockerti* Schmidt, 1994, ♀ (1), juv. ♂ (2) (car. 3.9, 7.8, 15.0) (NMPC P6d-61/2018), juv. (3) (car. 3.2) (NMPC P6d-61/2018), Peru, Loreto; *Tmesiphantes hypogeus* Bertani et al., 2013, ♀ holotype (MNRJ 04357), Brazil, Bahía, Andaraí, Igatu, Gruna da Parede Vermelha;

*Vitalius paranaensis* Bertani, 2001, ♂ (1) (NMPC P6d-61/2018), juv. (2) (car. 5.2) (NMPC P6d-61/2018), unknown origin.

Voucher material of Theraphosinae is deposited in the NMPC under inventory number P6d-61/2018.

Abbreviations: INPA = Instituto Nacional de Pesquisas da Amazônia, Brazil; NHMW = Natuhistorisches Museum Wien, Austria; MNRJ = Museu Nacional do Rio de Janeiro, Brazil; MUSM = Museo de Historia Natural, Lima, Peru; NMPC = National Museum, Natural History Department, Praha, Czech Republic; SMFD = Senckenberg Museum Frankfurt, Germany; UNAM = Universidad Autónoma de México, Facultad de Ciencias, Laboratorio de Acarología; juv. = juvenile specimen; car. = length of carapace. The number in parenthesis following the scientific name of particular species refers to the relevant specimen in the group.
